# Carbon‐Negative Ammonia Production from the Air

**DOI:** 10.1002/anie.202423934

**Published:** 2025-07-31

**Authors:** Dingqi Wang, Xue Yan, Jining Guo, Longbing Qu, Chao Wu, Jefferson Zhe Liu, Ali Zavabeti, Gang Kevin Li

**Affiliations:** ^1^ Department of Chemical Engineering University of Melbourne Parkville Victoria 3010 Australia; ^2^ Department of Mechanical Engineering University of Melbourne Parkville Victoria 3010 Australia; ^3^ Department of Chemical Engineering RMIT University Melbourne Victoria 3001 Australia

**Keywords:** Carbon‐negative ammonia synthesis, Carbon storage, Energy saving direct air capture, Green ammonia production, Nitrogen fixation

## Abstract

Ammonia holds paramount importance as a fundamental chemical commodity for large‐scale production of fertilisers and hydrogen carriers. The conventional Haber–Bosch process relies heavily on fossil fuels, making ammonia synthesis a significant contributor to greenhouse gas emissions. Here, we show a carbon‐negative ammonia synthesis process that not only produces ammonia from the air but also directly captures the atmospheric CO_2_ (DAC). This process uses lithium as a mediator to cleave the nitrogen gas via a nitridation reaction at ambient pressure below 80 °C. Exposing the nitride to moisture naturally yields ammonia. The co‐produced lithium hydroxide intermediate allows for spontaneous absorption of CO_2_ with an exceptional capacity of 18.4 mmol CO_2_/g. The resultant lithium carbonate is electrolysed at ambient conditions to recycle the metallic lithium and release high‐purity gaseous CO_2_. This proof‐of‐concept of ammonia production from air coupled with DAC paves a new way for the development of sustainable and negative‐emission technologies.

## Graphical abstract

Our work demonstrates a carbon‐negative ammonia synthesis process that not only produces NH_3_ from air but also directly captures the atmospheric CO_2_ (DAC), which achieves low‐cost DAC process with exceptional CO_2_ capacity and free ammonia production. This paves a new way in the development of sustainable and negative‐emission technologies.

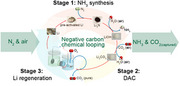

## Introduction

Current chemical engineering processes that generate significant CO_2_ emissions necessitate critical re‐engineering for future technological growth and sustainable adoption. Ammonia, a cornerstone of the global economy, serves vital roles as a fertiliser feedstock, an industrial and household chemical, and a chemical precursor, with additional potential as a future carbon‐free fuel and hydrogen storage molecule.^[^ ^1^, ^2^ ^]^ Approximately 170 million metric tonnes of ammonia are produced worldwide annually, with production projected to increase by 2.3% per year.^[^ ^3^ ^]^ Despite ammonia being widely used and considered a potential zero‐carbon molecule critical to the second chemical revolution, the current industry large‐scale ammonia production is the Haber‐Bosch process operating at high pressure and high temperature, requires fossil fuel stock as a feasible energy source and on‐site hydrogen sources through the hydrocarbon reforming process. Ammonia production consumes 1%–2% of the world's annual energy supply and is responsible for more than 1.6% of global carbon emissions.^[^ ^4^ ^]^

To date, numerous innovative methods and strategies have been employed to produce ammonia more economically and in an environmentally friendly manner.^[^ ^5^ ^]^ While extensive works have been focused on the electrochemical synthesis of ammonia directly from air/N_2_ and water, until now, the Faraday efficiency (FE) in aqueous systems remains mostly limited to around 30%.^[^ ^6^, ^7^, ^8^, ^9^, ^10^, ^11^, ^12^, ^13^, ^14^, ^15^, ^16^ ^]^ Some other recent studies have explored the use of Li as a mediator, offering heightened reactivity with nitrogen under standard atmospheric conditions to break the triple bond of nitrogen and transform it into nitride. Additionally, recent research works demonstrate that achieving high Faraday efficiency in the lithium‐mediated nitrogen reduction reaction (Li‐NRR) system involves the use of additional high‐cost chemicals.^[^ ^17^, ^18^, ^19^, ^20^, ^21^, ^22^, ^23^, ^24^, ^25^, ^26^, ^27^, ^28^ ^]^ For example, alcohol is frequently employed in the electrolyte as a sacrificial proton carrier, which is continuously consumed and supplied by the electrochemical oxidation of H_2_ to produce alcohol.

On the other hand, given the pressing need to reduce the greenhouse gas CO_2_ emissions in every aspect of anthropogenic activities,^[^ ^29^, ^30^ ^]^ negative emission technologies play a pivotal role in slowing down or even reversing the concentration of atmospheric CO_2_ and enabling the sustainable production of carbon source essential to the chemical industry when fossil fuels phase out in the projected future. Direct air capture of CO_2_ (DAC) represents a collection of negative emission technologies based on various separation principles.^[^ ^31^, ^32^, ^33^, ^34^ ^]^ One major challenge of DAC is the high energy required for the regeneration of the carbon capture agent. Very recently, DAC plus integrated catalytic reduction of CO_2_ has gained much attention due to the ability to waive the regeneration step.^[^ ^35^, ^36^, ^37^ ^]^ However, how to integrate DAC with chemical engineering processes, particularly ammonia synthesis, remains largely unexplored to date, posing a significant opportunity/challenge in revolutionising process engineering.

In this study, we successfully unite an innovative ammonia (NH_3_) synthesis process with DAC to achieve carbon‐negative ammonia production from the air. This advanced process features a low energy and low waste‐generating chemical‐electrochemical Li‐assisted cycle. The cycle stages entail ammonia synthesis from the air and DAC, followed by Li regeneration. After the nitridation of Li, the produced Li_3_N naturally reacted with moisture in the ambient air, generating ammonia and lithium hydroxide (LiOH). Then the ammonia is recovered through absorption into liquid water. Subsequently, LiOH efficiently captured CO_2_ directly from the air (∼400 ppm), forming lithium carbonate (Li_2_CO_3_). The products underwent acidification to liberate CO_2_ and yield the necessary electrolyte for the Li metal recovery process. Finally, lithium ions (Li^+^) were electrochemically converted to metallic lithium (Li^0^), and the acid was regenerated, thus bringing the cycle to a close loop.

Given that the moderate energy requirements for chemical reactions and electrochemical recovery at near or room temperature can be met through renewable energy sources and that the DAC stage effectively removes CO_2_ from the atmosphere, the overall process operates as a carbon net‐negative system. This methodology represents a significant advancement in negative emission technology for the sustainable production of ammonia. Meanwhile, our results suggest that the integration of multiple chemical engineering processes is a promising, effective, and energy‐efficient strategy to address the long‐standing problem in the chemical synthesis processes.

## Results and Discussion

**Figure 1:**
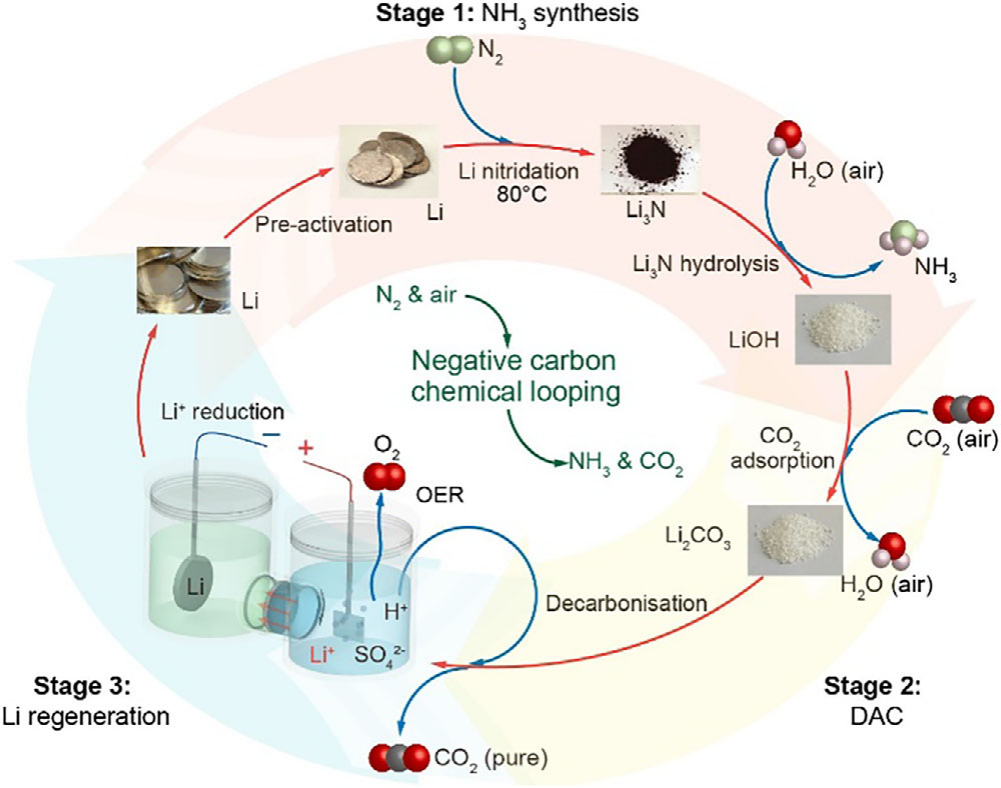
The schematic diagram of the three‐stage cyclic process. The overall process entails ambient pressure lithium‐assisted integrated production of NH_3_, DAC of CO_2_, and Li regeneration, respectively. Stage 1 demonstrates the synthesis and production of NH_3_. Stage 2 shows DAC of CO_2_ producing Li_2_CO_3_. Stage 3 elucidates Li recovery. To close the loop, Li is recovered through an electrodeposition regeneration electrochemical reaction. Stage 1 includes a nitrogen fixation reaction temperature of 80 °C, with all other stages occurring at room temperature and ambient pressure. The moderate energy requirements of the stages feature sustainable processes compatible with renewable energy sources. This characteristic makes it well‐suited for decentralised production and transportation.

As shown in schematic Figure 1, we demonstrated a three‐stage process of Li‐assisted chemical loop including: i) **NH_3_ synthesis**. Li pre‐activation and consequent nitrogen fixation (Equation 1), and the hydrolysis of Li_3_N with the moisture in the air to produce ammonia and an alkaline material LiOH (Equation 2). ii) **DAC**. Absorption of atmospheric CO_2_ by the alkalines (Equation 3) followed by acid decarbonisation to produce highly concentrated CO_2_ (Equation. 4). iii) **Li regeneration** by Li electrodeposition in a hybrid aqueous/Li‐ion conducting glass‐ceramics (LICGC)/organic electrolytic cell together with acid reproduction (Equations 5 and 6). The overall reaction process can be summarised in Equation 7, with air components including N_2_, H_2_O, and CO_2_ as the only feedstock, pure CO_2_, O_2_ and NH_3_ as the main products, and renewable power as the energy input, demonstrating a chemical loop with Li serving as an integrated catalyst that is not consumed.

The reactions in each step consist of the following equations:

Stage 1:
(1) $$6Li_{\text{(pre-activated)}}+\;N_{2}\to \text{2L}i_{3}N$$
(2) $$2Li_{3}N+6H_{2}O_{\left(\text{from air}\right)}\to 6\text{LiOH}+2NH_{3}$$
Stage 2:
(3) $$6\text{LiOH}+3CO_{2\;(400\text{ppm from air})}\to 3Li_{2}CO_{3}+3H_{2}O$$
(4) $$3Li_{2}CO_{3}+3H_{2}SO_{4}\to 3Li_{2}SO_{4}+3H_{2}O+3CO_{2\;\text{(pure)}}$$
Stage 3:
(5) $$\text{Cathode}:\;6{\mathrm{Li}}^{+}+\;6e^{-}\to 6\text{Li},\;\text{in}\;1M\;\text{LiCl}O_{4}/\text{PC}$$
(6) $$\text{Anode}:\;3H_{2}O\;-6e^{-}\to 3/2O_{2}+6H^{+},\;\text{in}\;1M\;Li_{2}{\mathrm{SO}}_{4}/H_{2}O$$
Overall:
(7) $$\begin{array}{cc} & N_{2}+3H_{2}O_{\left(\text{from air}\right)}+3CO_{2(400\text{ppm from air})}\\ & \;\to 3CO_{2(\text{pure})}+3/2O_{2}+2NH_{3} \end{array}$$

### Stage 1. NH_3_ Synthesis

**Figure 2:**
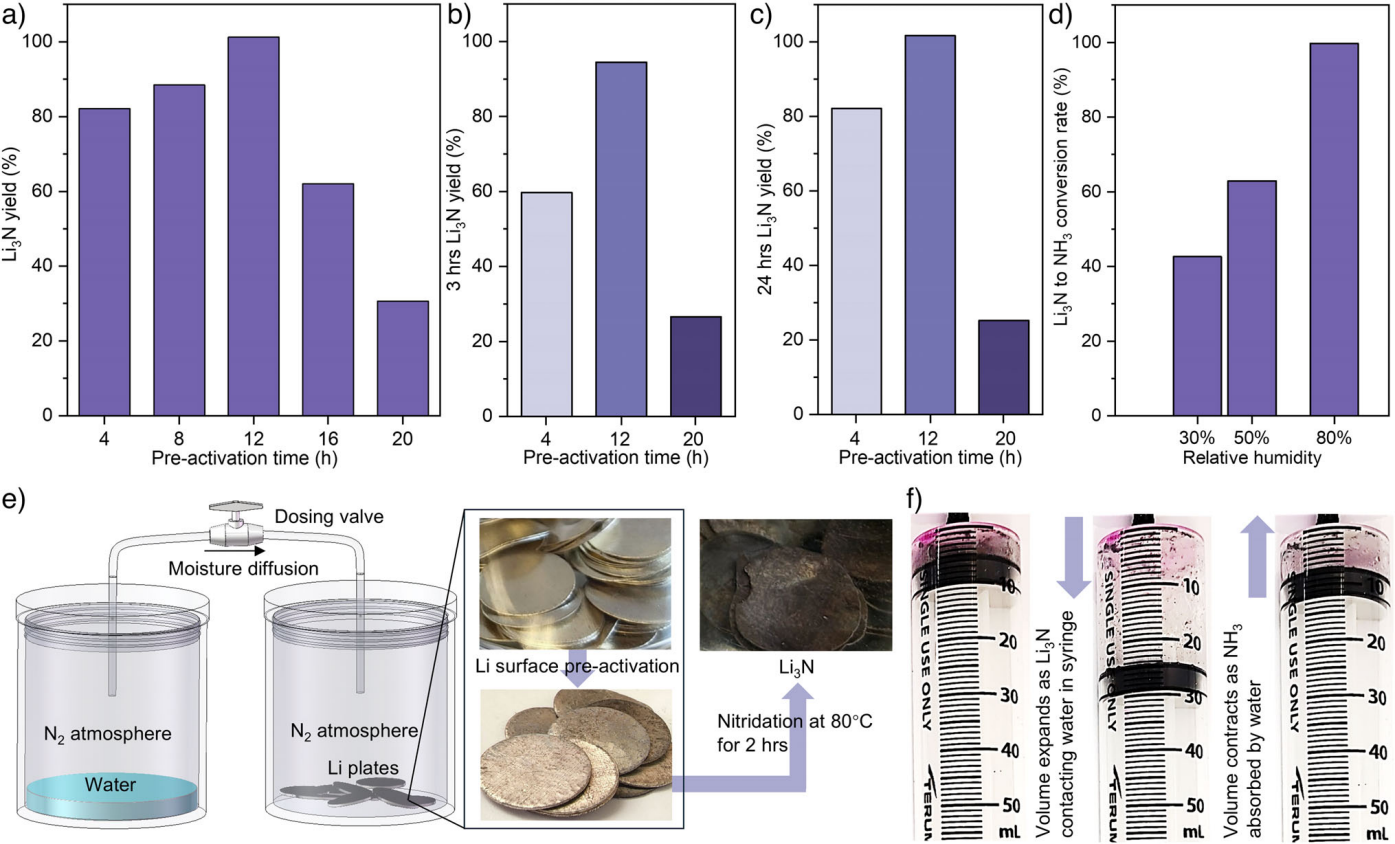
Pre‐activation, nitrogen fixation on Li plate and the reaction between moisture and Li_3_N. a) Li_3_N yield under different pre‐activated times, all samples are then treated with N_2_ for 24 h at 80 °C after the pre‐activation. b) Li_3_N yield with different pre‐activated times and N_2_ treatment time of 3 h and c) 24 h at 80 °C. d) Conversion efficiency of commercial Li_3_N to ammonia under 2.5 L min^−1^ air flow and different relative humidities with the duration of 24 h. e) schematic representation of Li surface pre‐activation process by controlled moisture diffusion under N_2_ atmosphere. Optical photos show the surface of Li plate before, after pre‐activation and after nitridation. f) the exothermic hydrolysis reaction of Li_3_N, releases NH_3_ expanding the syringe volume. The NH_3_ is then dissolved and captured in H_2_O (phenolphthalein added as pH indicator), consequently contracting the syringe volume (Videos S1 and S2, detailed information in Experimental procedures and Supporting Information Notes 2).

Bulk Li remains stable under dry nitrogen for several months. As shown in Figure S1a, Li plates show a negligible colour change under dry N_2_ after 2 months. Interestingly, this constraint can be eliminated with pre‐exposure of the Li surface to moisture. This pre‐activation mechanism has been demonstrated to enhance the post‐nitridation reaction of Li metal^[^ ^38^ ^]^ (Figure S2). Therefore, activated Li can be used effectively during the nitridation reaction, and the nitridation can be accomplished under low temperatures (<100 °C). Without incorporating the pre‐activation step, the Li surface remained shiny under the nitridation experimental condition (Figure S1b). We incorporated this mechanism as an essential “pre‐activation” step prior to the nitridation reaction step with control for the moisture exposure to Li (more detail in Supplementary information–Preparation of the pre‐activated lithium samples section). The controlled dosing of moisture uses N_2_ as the carrier gas (Figure S3). The duration of the pre‐activation step is then optimised according to the nitrogen fixation reaction, as elucidated in Figure 2a,b,d. The final products were reacted with H_2_O to measure the ammonia concentration and calculate the nitrogen fixation reaction specified as conversion efficiency (Figure 2a,b,d and Equation 1). With a pre‐activation of 12 h, a maximum conversion efficiency of 99.9% was achieved. This result indicates that the controlled introduction of moisture consumes less than 0.1% of lithium (based on the mass balance of NH_3_ and Li). The quantitative measurements were performed using the amount of generated NH_3_ measured by nuclear magnetic resonance (NMR) spectroscopy and confirmed with Ultraviolet‐Visible (UV–vis) Spectroscopy (Figures S4 and S5).

To analyse the conversion efficiency and kinetics of preactivated Li to Li_3_N, the preactivated Li was exposed to N_2_ with different durations, up to 24 h, which was used to calculate the conversion rate of Li to Li_3_N. Accordingly, the optimised pre‐activation time of 12 h was obtained (Figure 2a). To analyse the conversion kinetics of Li to Li_3_N, the pre‐activated Li metal was consequently exposed to N_2_ at 80 °C for different durations of 3 and 24 h, achieving ∼94.4% and ∼100% conversion rate of Li to Li_3_N (Figure 2b,c), respectively. Common practices for Li_3_N synthesis from Li and nitrogen involve an operating temperature range of 180–250 °C. ^[^ ^39^, ^40^ ^]^ However, processing melted lithium poses safety concerns, and it would be desirable to operate the reaction of lithium with nitrogen at a temperature below its melting point. The process demonstrated here provides a pathway to pre‐activate Li with moisture, leading to an energy‐effective process on the nitridation reaction, which can be achieved with nearly 100% conversion efficiency working under lower temperatures (80 °C) and ambient pressure.

To obtain NH_3_ from Li_3_N, liquid water was reacted with Li_3_N with ease. The hydrolysis reaction is spontaneously, leading to the rapid and substantial liberation of NH_3_ gas (Figure 2f; Videos S1 and S2, Supporting Information Notes 2). Li_3_N features high reactivity with air moisture under ambient conditions, producing LiOH and releasing NH_3_. Therefore, the moisture directly from the air is utilised as the reactant to release NH_3_ from Li_3_N (Figures 1 and 2c), producing LiOH. The produced LiOH naturally captures and removes CO_2_ from the air, resulting in the formation of Li_2_CO_3_. Meanwhile, the generated NH_3_ is efficiently captured through absorption into liquid water, enabling its separation from the carrier air. Thus, NH_3_ synthesis in air can be coupled with DAC technology, wherein Li_3_N reacts with air moisture to release NH_3_, and the resulting LiOH products capture CO_2_ from the air. In addition, direct exposure to air moisture is also superior to adding liquid water to Li_3_N due to the highly exothermic reaction and the high water‐solubility for both Li^+^ ions and NH_3_, which makes the separation and recycling processes challenging. Hence, the devised moderate condition for producing ammonia allows for controlled NH_3_ production of while capturing CO_2_.

**Figure 3:**
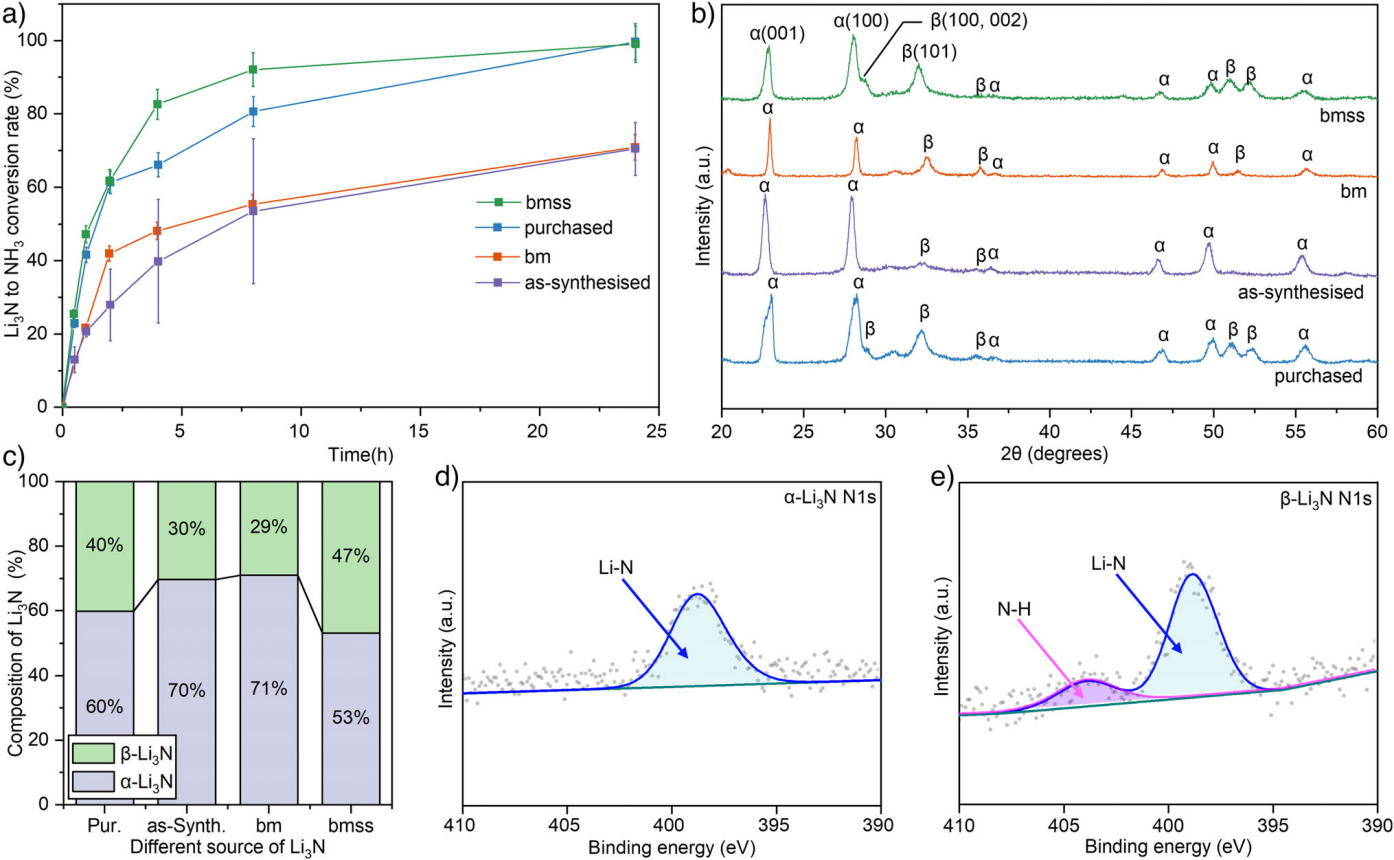
Li_3_N to NH_3_ conversion rate in air moisture and the characterisation of Li_3_N. a) The conversion rate from different preparation methods of Li_3_N to NH_3_, the data shown as the means ± SD (*n* = 3). b) The XRD results for Li_3_N prepared using different methods. c) The composition of Li_3_N obtained from different preparation methods, elucidating the proportion of α‐Li_3_N and β‐Li_3_N. (purchased: commercial lithium nitride; as‐synthesised: as‐synthesised lithium nitride without ball milling and ground by mortar and pestle; bm: as‐synthesised sample including ball milling with agate balls; bmss: as‐synthesised sample including ball milling with stainless steel balls.) d) N1s XPS spectra for as‐synthesised Li_3_N and e) N1s XPS spectra for the bmss Li_3_N sample. The bmss sample underwent partial conversion to NH_3_ with an appearance of a small N─H peak. This is due to the sample instability in the air during transportation and handling for XPS measurements.

Figures 2d and S6 shows the conversion rate for commercial and synthesised Li_3_N from Li_3_N to NH_3_ under varying relative humidities. An increase in humidity clearly results in a higher conversion rate. Enhanced reaction kinetics are especially shown for Li_3_N with increased adsorbed water molecules.^[^ ^41^ ^]^ Complete conversion of Li_3_N to LiOH (Figures 2c and 3a) is also consistent with findings from previous reports that Li_3_N is unstable under high humidity conditions.^[^ ^42^ ^]^

The majority of previous works have focused on commercial Li_3_N to study its moisture stability, while the stability difference between different Li_3_N crystal structures and morphologies in response to moisture has remained under‐explored. Figure 3a shows the conversion kinetics of Li_3_N to NH_3_ using different Li_3_N synthesis methods. The as‐synthesised and bm Li_3_N resulted in a relatively low conversion rate of ∼60% while the bmss Li_3_N achieve a conversion rate comparable to that of the purchased Li_3_N with over 99% conversion to ammonia within 24 h. The bm and bmss Li_3_N was processed using ball‐milling with agate and stainless‐steel balls from as‐synthesised Li_3_N, respectively. The ammonia conversion kinetics of Li_3_N obtained from different routes show significant differences, as presented in Figure 3a. This difference may be attributed to the change in particle size and crystal structure. The powder particle sizes were characterised using scanning electron microscope (SEM) image processing. Considering consistent particle size between the bm and bmss samples (Figure S7), a relatively similar reaction kinetics was anticipated. However, the bm kinetics anomaly features significantly lower Li_3_N to ammonia kinetics than that of the bmss sample. Understanding the mechanism of the hydrolysis of Li_3_N is crucial for improving the kinetics and, consequently, the reaction yield. Therefore, we further investigated the crystal structure and reaction mechanism of different Li_3_N samples (Figures S7 and S8). After analysing ∼70 Li_3_N specimen high‐resolution crystal grains by high‐resolution transmission electron microscopy (HRTEM) (Figures S8 and S9), the proportion of the beta phase was found to be higher than the alpha phase in the bmss sample than that of the as‐synthesized sample. This is in agreement with the XRD obtained ratio for different phase of Li_3_N presented in Figure 3b,c. The as‐synthesised and bmss samples were also characterised by XPS (Figure 3d,e). The peak located at 398 eV consists of the N1s obtained from Li_3_N.^[^ ^43^ ^]^ Figure 3a shows that the sample with a higher beta phase ratio exhibits higher reactivity. Because the samples were momentarily exposed to air prior to sample loading under vacuum for XPS characterisation, the samples were partially decomposed to form an intermediate N─H bond located at 403 eV. This confirms the hydrolysis of β‐Li_3_N with air moisture featuring high kinetics (Figure 3a).

**Figure 4:**
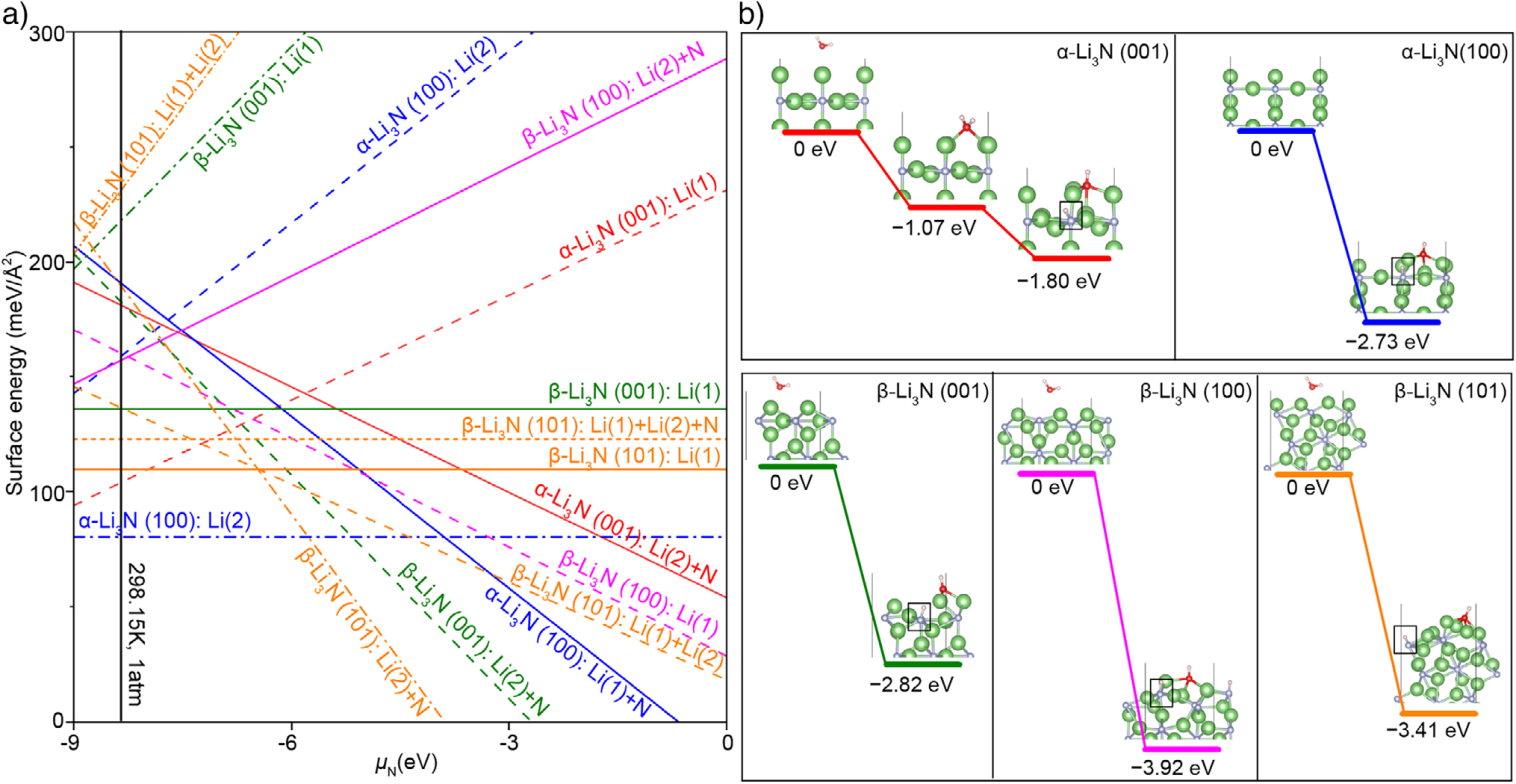
Density Functional Theory (DFT) calculation about the reactivity of α/β‐Li_3_N in H_2_O decomposition. a) Calculated surface energy (γ) of various α/β‐Li_3_N surfaces plotted against the nitrogen chemical potential (µ_N_) variation. These surfaces correspond to those identified in XRD analyses. The black solid line represents the experimental conditions of 1 standard atmosphere (atm) pressure at a temperature of 298.15 K. b) The energy profile of H_2_O adsorption and decomposition on the most stable α/β‐Li_3_N surfaces at 1 atm at 298.15 K. The selected most energetically favourable surfaces are shown in Figure S10. The ground state for each energy profile is the unadsorbed state. The black rectangle highlights the decomposed H.

We conducted density function theory (DFT) calculations to gain fundamental insight into this hydrolysis reaction mechanism and explored experimental observations of reaction kinetics differences between our samples. The DFT calculations were employed to investigate the hydrolysis reaction kinetics. X‐ray diffraction (XRD) (Figures 3b and S10) identified crystal facet orientation characteristics, predominantly exposing the (001) and (100) α‐Li_3_N surfaces while exposing (001), (100), and (101) β‐Li_3_N surfaces. These low‐index surface facets may terminate with distinct configurations of Li(1), Li(2), and N atoms and exhibit different stability and reactivity. Therefore, we calculated these surface energies to identify the relatively stable termination among these low‐index surfaces (Details in Supporting Information Experimental procedures section). Previous studies have suggested that Li(1) forms a Li layer, whereas Li(2) forms a Li‐N layer with N.^[^ ^44^ ^]^ Refining our approach due to spatial complexities in β‐Li_3_N, we identified Li(2) and N atoms within the same plane, forming three equivalent Li─N bonds (Figure S10a). Figure S10b illustrates all possible surface terminations among these surfaces. Figure 4a shows the surface energies of various α‐Li_3_N and β‐Li_3_N surfaces as a function of nitrogen chemical potential, where lower values of surface energy indicate higher stability. Under the experimental conditions marked by the solid black line, the comparatively stable surface terminations have been identified as follows: α‐Li_3_N (001) with Li(1) termination, α‐Li_3_N (100) with Li(2) termination, β‐Li_3_N (001) with Li(1) termination, β‐Li_3_N (100) with Li(2) termination, and β‐Li_3_N (101) with N termination (Figure S11). In the following, the term “stable surface” refers to surface terminations that exhibit the lowest surface energy among those considered for α‐Li_3_N and β‐Li_3_N in this study.

We further explored the adsorption and decomposition of H_2_O on these stable surfaces to simulate the interaction of Li_3_N with atmospheric H_2_O. Figure 4b displays the configurations and energy profiles for H_2_O adsorption and decomposition on these stable surfaces. It is important to note that two types of the β‐Li_3_N (100) surface showed very close surface energies (differing by only 3 meV Å^−2^). Therefore, we also analysed the H_2_O adsorption and decomposition on the β‐Li_3_N (100) surface with Li(1) termination (Figure S12). As depicted in Figure 4b, H_2_O adsorption and decomposition occur as two separate processes on the α‐Li_3_N (001) surface, releases energy of 1.8 eV. In contrast, on the other surfaces, H_2_O decomposes spontaneously upon adsorption, accompanied by a decrease in energy. For the comparatively stable (101) β‐Li_3_N surfaces, the energy reductions are more pronounced, up to 3.41 eV. The less stable β‐Li_3_N (100) surface also exhibits spontaneous H_2_O decomposition upon adsorption, with an energy decrease of 3.93 eV (Figure S12).

To elucidate the observed spontaneous decomposition and significant energy reductions during H_2_O interaction with various surfaces, we conducted a Bader charge analysis to assess the chemical reactivity of Li atoms on these surfaces. Table S1 summarises the electron loss from Li(1) and Li(2) atoms on the five surfaces before H_2_O adsorption, with the electron count of an isolated neutral Li atom serving as the reference for comparison. The Li atoms on the α‐Li_3_N (001) surface have lower electron loss, indicating weaker reactivity. On the other four surfaces, Li atoms lose more electrons and acquire a highly positive charges, suggesting a high reactivity that could promote the spontaneous decomposition of H_2_O on these four surfaces.

Our calculations indicate that the reaction between Li_3_N and H_2_O on all examined surfaces leads to energy release, facilitating the formation of LiOH and NH_3_. However, the catalytic reactivity of different surfaces significantly influences the reaction rates. Specifically, the relatively stable surfaces of the β‐phase exhibit superior activity in the decomposition of Li_3_N via H_2_O compared to the α‐phase surfaces. Among the α‐phase surfaces, only the α‐001 surface supports the spontaneous hydrolysis of Li_3_N. Overall, the hydrolysis on β‐phase surfaces results in a greater release of energy than on α‐phase surfaces. This is consistent with the accelerated reaction rates of β‐phase surfaces observed in experiments.

### Stage 2. DAC

**Figure 5:**
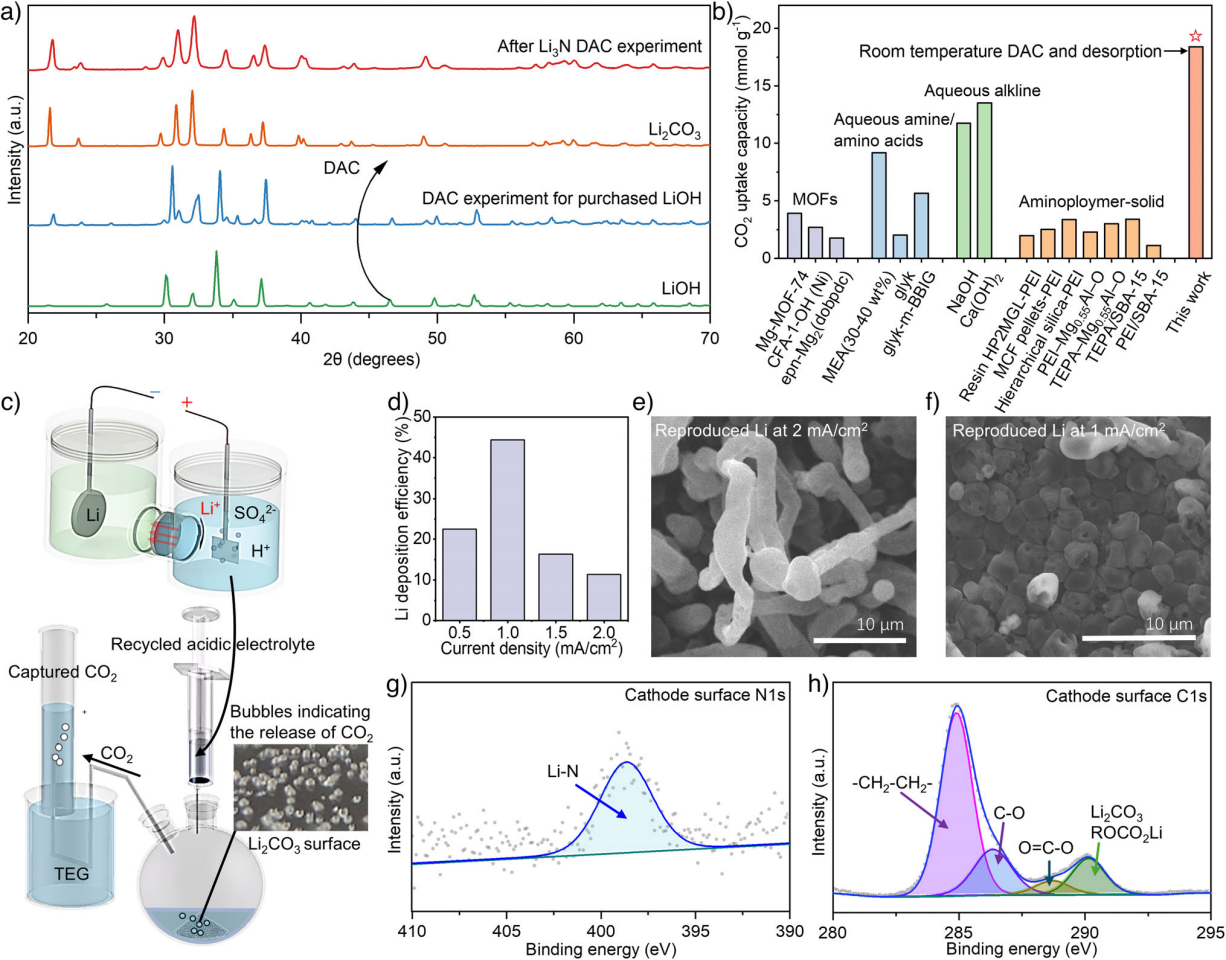
CO_2_ desorption and Li regeneration. a) XRD measurements of the reaction products between Li_3_N and moisture air, including reference purchased powder XRD of Li_2_CO_3_ and control experiment for purchased LiOH. b) The comparison of DAC CO_2_ uptake in this work and various typical and recently developed DAC agents. c) Schematic of the drainage gas collection process, in the Li regeneration cell, the acidic electrolyte is generated at the anode and subsequently injected into a flask contains Li_2_CO_3_, the product of DAC. The release of CO_2_ gas is indicated by the formation of bubbles at the bottom of the flask. The CO_2_ is collected via the drainage gas method, as shown by the direction of the arrows, and finally captured as pure CO_2_. d) Regenerated Li was used to reproduce ammonia in the next chemical loop cycle. The produced ammonia was measured to calculate the efficiency of the Li regeneration in the previous step. The bar chart shows the calculated realistic Li regeneration efficiency when it was recycled for ammonia synthesis. The steady‐state current was used for FE calculation, and initial SEI‐formation‐induced transient states were excluded. The regeneration efficiency varied with current densities, as shown. e) SEM image of the cathode electrode surface after 1 h, with current densities of 2 mA cm^−2^ and f) 1 mA cm^−2^. g) N 1s XPS peak after nitridation of recycled Li and (h) C 1s XPS spectra from the cathode electrode surface after electrolysis showing the development of characteristic peaks of solid electrolyte interphase (SEI).

In addition to the ammonia production reaction that occurs naturally in air, solid LiOH is produced simultaneously (Equation 2). Strikingly, as the ammonia reaction proceeded, the LiOH products reacted with CO_2_ directly from the air, forming Li_2_CO_3_ (Equation 3, detailed in Experimental procedures). XRD results shown in Figure 5a elucidate peaks corresponding to Li_2_CO_3_, confirming the conversion of LiOH. A control experiment (Figure 5a) also confirms that purchased LiOH naturally converts to Li_2_CO_3_ during exposure to air for 24 h with slower kinetics compared to that of experimentally obtained LiOH. Subsequently, CO_2_ can be released by adding H_2_SO_4_, which produces an aqueous electrolyte containing Li ions (Equation 4). Moreover, the H_2_SO_4_ used in this step is recycled from the anode reaction in Stage 3 (Schematic Figure 1), indicating the design of an environmentally friendly process for CO_2_ capture and release. The released CO_2_ gas is collected and analysed using GC equipment, which contains 99.9% CO_2_ (Figure S13).

Meanwhile, the material featured a relatively high weight capacity for DAC. A calculated CO_2_ uptake of 18.4 mmol CO_2_ g^−1^ was achieved at a productivity of 1.53 mmol h^−1^ g^−1^ (Supporting Information Notes 3), considering LiOH as the DAC material. Figures 5b and S14; Table S2 illustrate the CO_2_ uptake for various typical and recently developed DAC technologies, including chemical absorption with alkaline or amino acid‐based aqueous solution, amino polymer with silica or resin and adsorption with Metal‐Organic Frameworks (MOFs).^[^ ^33^, ^34^, ^45^, ^46^, ^47^, ^48^, ^49^, ^50^, ^51^, ^52^, ^53^, ^54^, ^55^ ^]^ Compared to the high‐performance aqueous alkaline DAC systems of Ca(OH)_2_ with 13.5 mmol CO_2_ g^−1^, the synthesised material shows more than 36% enhancement of CO_2_ adsorption. Moreover, compared with alkaline and amines as benchmarks of DAC, the method presented does not require aqueous media, supporting materials, or elevated temperatures for CO_2_ capture and release, respectively.

### Stage 3. Lithium Regeneration

To close the cycle, the mediated Li that was used for the NH_3_ synthesis and DAC was regenerated on the cathode surface through the electrochemical conversion of Li^+^ ions to metallic Li. The catholyte is composed of 1M LiClO_4_ in propylene carbonate (PC) solvent. At the anode, the reaction is engineered and devised so that the oxygen evolution reaction acts as an electron donor reaction while also generating sulfuric acid due to the production of proton at the anode. The experimentally produced H_2_SO_4_ subsequently was used to release CO_2_, which in turn produced Li_2_SO_4_, serving as a lithium salt in anolyte (Figure 5c). This developed green chemical looping method for the regeneration of acid and anolyte are demonstrated in Figure 1 with a blue circular arrow. To prevent the mixing of deposited Li with aqueous anolyte, the transport of produced proton to catholyte and the free transport of lithium ions from anolyte to catholyte, Lithium‐Ion Conducting Glass‐Ceramics (LICGC) is utilised as a separator (Figure S15).

The electrochemical reactions are shown below:

(8) $$\text{Cathode reaction}:{\text{Li}}^{+}+e^{-}\to \text{Li}$$

(9) $$\text{Anode reaction}:H_{2}O-2e^{-}\to 2H^{+}+1/2O_{2}$$

(10) $$\text{Overall reaction}:2{\text{Li}}^{+}+H_{2}O\to 2H^{+}+1/2O_{2}+2\text{Li}$$

The electrochemical performance of this cell was evaluated by LSV (Figure S16), revealing a linear curve when the voltage is greater than −5.3 V. This is consistent with the literature, indicating the high and dominant ohmic resistance from the ceramic membrane.^[^ ^5^ ^]^ The LSV was initiated based on the theoretical value of −3.86 V, considering the oxygen evolution potential at 0.82 V in aqueous Li_2_SO_4_ and with reduction potential of Li ions at −3.04 V.

Determining the FE of this reaction is challenging due to the high reactivity of newly deposited Li with air, rendering the quantification of metallic Li a complex process. One possible quantification approach is to utilise regenerated Li for the subsequent step of nitridation and ammonia release processes. Then by the quantification of ammonia, the amount of regenerated Li can be calculated, providing an FE representing the realistic efficiency of the designed chemical loop. Figure 5d illustrates the FE calculated under different current densities. The presented method for Li regeneration FE and current densities is comparable with highly regarded literature reports, yielding an FE of 44.4%.^[^ ^8^, ^22^, ^25^, ^56^, ^57^, ^58^, ^59^ ^]^ This includes technologies involving nitrogen reduction and hydrogen uptake to produce NH_3_. Our work presents an alternative to the use of H_2_, which typically requires water splitting or fossil fuels for its production.

SEM analysis of the deposited Li on the Cu plate reveals a highly formless structure with complex layers of dendrites while the current exceeds 2 mA cm^−2^ (Figure 5e). This indicates that the dendrite formation is challenging to avoid under high current density when using LiClO_4_/PC as the cathode electrolyte for lithium deposition. The high surface area of SEI and dendrites consumes “live” Li and decreases the efficiency. When the current density lowered to 1 mA cm^−2^, the surface of cathode exhibited a densely packed arrangement of uniform spheres or cylinders (Figure 5g). This surface configuration, where Li forms a denser SEI with reduced surface area, enhances the Faradaic efficiency. However, when current densities below 1 mA cm^−2^ are applied, voids are observed on the electrode surface, indicating suboptimal experimental conditions (Figure S18). The morphology from our electrochemical deposition of lithium metals is consistent with the literature.^[^ ^60^, ^61^, ^62^, ^63^ ^]^ XPS performed on the cathode surfaces (Figure 5g,h) after THF cleaning and after the lithium nitration process confirmed the presence of Li_3_N and a corresponding SEI containing Li_2_CO_3_ and ROCO_2_Li.^[^ ^5^ ^]^ The N1s peaks in the XPS spectrum were consistent with the Li_3_N XPS patterns, confirming the Li_3_N formation on the cathode as a precursor of ammonia. The presence of SEI limits the Li regeneration efficiency. The Li recovery efficiency is also confirmed through inductively coupled plasma‐optical emission spectroscopy (ICP‐OES) (Figure S19). The results show that all remaining Li‐containing compounds on the cathode surface after the reaction can be almost completely recycled by dissolving them in aqueous solution and using them as an anode electrolyte in the cycled loop.

Specifically, the synthesis rate of NH_3_ was quantified. By keeping 1 kg of Li element in the loop, an overall production rate of approximately 150 kg of NH_3_ per annum can be achieved (Figure S20). The synthesis rate of Li_3_N was determined to be 3.2 mmol h^−1^ g_Li_^−1^ while the subsequent NH_3_ release rate was 1.2 mmol h^−1^ g_Li3N_^−1^ (Supporting Information Notes 3). Compared with recent reports on NH_3_ synthesis from N_2_ in aqueous systems, this rate is considerably high (Table S3). Additionally, the calculated regeneration rate of metallic lithium in Stage 3 was 4.5 µmol h^−1^ cm^−2^. Notably, the reaction rates for nitridation, NH_3_ release, and direct air capture (DAC) are all within the same order of magnitude. To achieve a rate‐matching electrolysis step, the electrode surface area would need to be scaled to approximately 1000 cm^2^ (roughly equivalent to the area of an A4 sheet) per gram of lithium.

**Figure 6:**
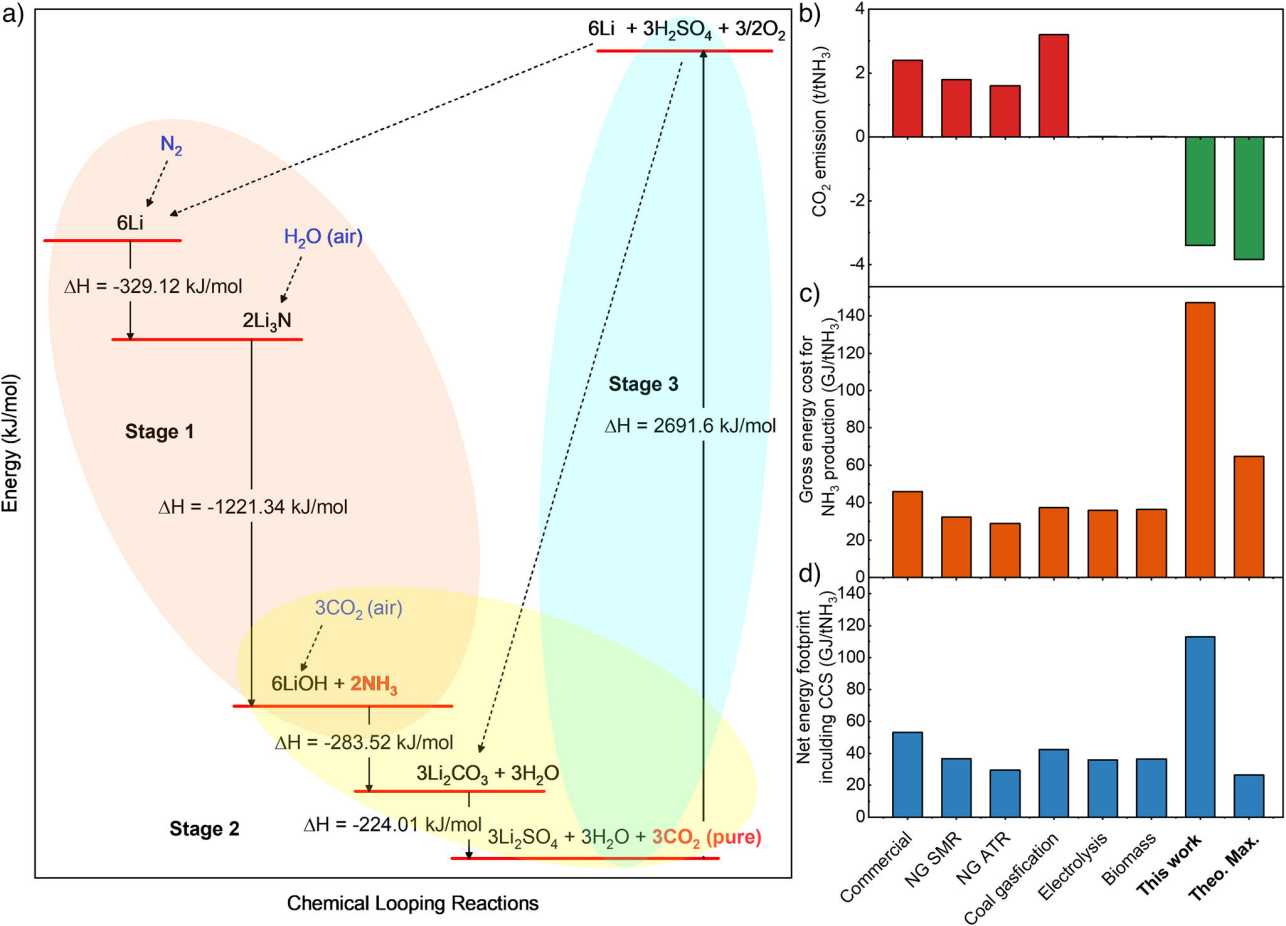
Energy analysis for NH_3_ production and carbon capture and storage (CCS). a) Energy profiles of the three‐stage Li‐assisted chemical loop. For each stage of the chemical looping process, the enthalpy is calculated based on the experimentally derived standard enthalpy of formation. The key Li compounds are labelled for each step, the dashed arrows indicate other compounds involved in the loop, the substances in blue represent chemicals supplied externally to the loop and the pure CO_2_ and NH_3_ in red font represent the products harvested from the loop. A more detailed figure is included in Figure S21. b) CO_2_ emissions of different NH_3_ production processes. c) Gross energy cost for different NH_3_ production processes. d) Net energy footprint including CCS to cover the CO_2_ emission for different NH_3_ production processes. (Commercial: current commercial NH_3_ production in average, others are the best available technology; NG: natural gas; SMR: steam methane reforming; ATR: auto‐thermal reforming; Theo. Max.: the theoretical maximum CO_2_ capture amount and maximum efficiency in this work.)

In our devised green ammonia production process, all the reaction steps except Li recycling are spontaneous and performed at or near room temperature. Remarkably, sulfuric acid is produced during the Li recycling process, which can be reused in CO_2_ desorption and consequently as an electrolyte in the electrodeposition of Li. Alkaline LiOH is also generated as a byproduct in the loop with exceptional CO_2_ uptake for the DAC process. The DAC process does not require additional energy for absorption and regeneration reactions, respectively (Figure 6a).^[^ ^64^ ^]^ In contrast, conventional DAC processes are energy intensive, necessitating significant energy for the regeneration of adsorbents or absorbents (e.g., by heating up to decompose and release CO_2_ or via pressure swing to desorption). The average energy consumption of DAC is approximately 10 GJ per ton of CO_2_.^[^ ^65^, ^66^ ^]^ In our scenario, 3.4 tons of CO_2_ are captured per ton of NH_3_ produced (Figure 6b), which indicates an energy saving of ∼34 GJ per ton of NH_3_ produced when coupled with DAC (Supporting Information Notes 4). Furthermore, with the potential improvements in the Faraday efficiency and DAC yield, we calculated the gross energy consumption and the net energy consumption when incorporating CCS (Figure 6c,d).^[^ ^67^ ^]^ The combined energy consumption for the integrated NH_3_ synthesis and DAC is lower than that for independent DAC on the basis of per unit mass of CO_2_ captured (Figure 6d). This result suggests our novel carbon negative ammonia synthesis technology can be also considered as a low‐cost DAC process with free ammonia production, promising transformational pathway for industrial applications.

## Conclusion

In conclusion, we successfully developed an innovative NH_3_ synthesis process with a combination of the DAC process to achieve carbon‐negative NH_3_ production from the air. Briefly, we produce NH_3_ and pure CO_2_ from the air and achieve lithium source recycling using the electrochemical lithium deposition methodology. This work demonstrated an excellent ammonia production rate of 4.5 µmol h^−1^ cm^−2^ and recorded high direct air CO_2_ uptake of 18.4 mmol CO_2_ g^−1^. In this newly developed carbon‐negative ammonia production methodology, Li is used as a nitrogen carrier to synthesize ammonia from N_2_. The direct moisture adsorption and reaction energies on different surface facets of produced α/β‐Li_3_N are fundamentally investigated, shedding light on enhanced kinetics observed from experimental hydrolysis of Li_3_N to produce NH_3_. All these chemical processes operate at room temperature and ambient pressure except the lithium nitridation, which was performed at 80 °C. The devised process demonstrates how the exorbitant synthesis conditions required for ammonia synthesis can be met under moderate conditions with a net negative CO_2_ emission. Compared with emerging electrochemical approaches for NH_3_ synthesis, the demonstrated process overcomes the competition of hydrogen evolution reaction, avoids the consumption of liquid water or sacrificial agents, and provides an effective pathway for DAC, which paves the way for NH_3_ synthesis from carbon neutral to carbon negative.

## Supporting Information

The authors have cited additional references within the Supporting Information.^[^ ^17^, ^18^, ^19^, ^20^, ^21^, ^22^, ^23^, ^24^, ^25^, ^26^, ^27^, ^28^, ^33^, ^34^, ^45^, ^48^, ^49^, ^50^, ^51^, ^52^, ^53^, ^54^, ^55^, ^66^, ^67^, ^68^, ^69^, ^70^, ^71^, ^72^, ^73^, ^74^, ^75^, ^76^, ^77^, ^78^, ^79^, ^80^, ^81^, ^82^, ^83^, ^84^, ^85^, ^86^, ^87^, ^88^, ^89^, ^90^, ^91^, ^92^ ^]^

## Conflict of Interests

The authors declare no conflict of interest.

## Supporting information



Supporting Information

Supporting Information

Supporting Information

## Data Availability

The data that support the findings of this study are available in the Supporting Information of this article.
