# Supplementary material for: Carbon‐Negative Ammonia Production from the Air
Source: Angew Chem Int Ed Engl. 2025 Jul 31;64(38):e202423934. doi: 10.1002/anie.202423934 (PMC12435413; doi:10.1002/anie.202423934)
Supplement: Supplementary file 1 — Supporting Information [file ANIE-64-e202423934-s002.docx]

**Supplementary Information**

**Carbon-Negative Ammonia Production from the Air**

Dingqi Wang^[a]^, Xue Yan^[b]^, Jining Guo^[a]^, Longbing Qu^[a]^, Chao Wu^[a]^, Jefferson Zhe Liu^[b]^, Ali Zavabeti^[a,c]^*, Gang Kevin Li^[a]^*

[a] Dingqi Wang, Dr. Longbing Qu, Dr. Chao Wu, Dr. Ali Zavabeti, Prof. Gang Kevin Li

Department of Chemical Engineering
University of Melbourne
Parkville, Victora 3010, Australia
E-mail: ali.zavabeti@unimelb.edu.au, li.g@unimelb.edu.au

[b] Dr. Xue Yan, Prof. Jefferson Zhe Liu
Department of Mechanical Engineering
University of Melbourne
Parkville, Victora 3010, Australia

[c] Dr. Ali Zavabeti
Department of Chemical Engineering
RMIT University
Melbourne, Victora 3001, Australia

**Table of Contents**

**Experimental procedures 3**

**Supplementary Notes 1-47**

**Tables S1-S411**

**Figures S1-S2315**

**Reference38**

**Experimental procedures**

**Materials and chemicals**

Lithium perchlorate (LiClO_4_, 99.99%), propylene carbonate (PC, anhydrous, 99.7%), lithium sulfate monohydrate (Li_2_SO_4_, 99.0%), sodium salicylate (NaC_7_H_5_O_3_, 99.5%), sodium hydroxide (NaOH, 98%), potassium sodium tartrate (KNaC_4_H_6_O_6_·4H_2_O, 99%), sodium nitroferricyanide dihydrate (Na_2_[Fe(CN)_5_NO] · 2H_2_O, 99%), sodium hypochlorite solution (NaOCl, available chlorine 4.00-4.99%), sulfuric acid (H_2_SO_4_, 98%), tetrahydrofuran (THF, anhydrous, 99.9%), phenolphthalein (C_20_H_14_O_4_, 98%) were all purchased from Sigma-Aldrich. Sheet of lithium-ion super ionic conductor (LICGC SP-01, Li₂O-Al₂O₃-SiO₂-P₂O₅-TiO₂, thickness 90 μm) was obtained from Ohara GmbH Inc, Pt electrode plate (10 × 10 × 0.1 mm) and copper plate (10 × 10 × 0.1 mm) was supplied from Yueci technology Co., Lithium chips (0.45 × 15.6 mm) were purchased from Gelon LIB Co.

**Preparation of the pre-activated lithium samples**

The pre-activated lithium samples are presented in Fig. 2b. Two flat bottom flasks were connected by a plastic tube with inner diameter of 3 mm, one of the flasks contained 20 mL of DI water (light blue colour) and the other was loaded with fresh and non-activated lithium thin plates (light grey colour). A dosing valve was placed in the connecting tubes to control the moisture exposure rate to the Li plates. The system was placed in N_2_ purged glove box under room temperature. The dosing was performed and when dark spots indicating a seed growth for nitridation process appeared on the Li plates the pre-activation was completed. The effect different activation times of 4 to 20 hours on nitridation kinetics are and presented in Fig 2.

**Preparation of Li_3_N**

After pre-activation step, the activated lithium samples were taken out of the glovebox and placed in another flask under continuous nitrogen gas flow of 60 mL/min and elevated temperature of 80 °C. The outlet of nitrogen gas was connected to air to retain the system near ambient pressure. During the reaction, the surface of the lithium plate changes to dark red color as shown in Figure S2. Then, the synthesized Li_3_N is manually grinded using mortar and pestle, and/or ball milled to produce powder morphologies. Ball milling was performed with a mass ratio of 1:20 between Li_3_N and stainless-steel balls. The ball milling was carried out at 500 rpm for 2 hours. (Planetary Ball Mill, PQ-N04, Across International) The ball mill tanks were packed inside nitrogen purged glovebox. The effect of ball mill and manual grinding on the unique crystal structure formation are analysed and presented in Fig. 3.

**Ammonia production and DAC**

The ~0.1 g of synthesised lithium nitride powder samples are then loaded into a 50 mL flask under a continuous airflow of 2.5 L/min under room temperature (25 °C) and certain relative humidity controlled by climate test chamber (DHT-100-40-P-SD, Shanghai Doaho Co., Ltd.) for 24 hours. Before we start the experiment, the chamber is reached the equilibrium conditions for 30 minutes. Air contains moisture and CO_2_ that can naturally produce ammonia and the solid by-products will capture CO_2_ from air. The produced ammonia is carried by air and then bubbled through an absorption column filled with 0.05 M sulfuric acid. The amount of ammonia absorbed by the sulfuric acid was used for production measurement. After the reaction was completed, the ammonia concentration in the solution was quantified using NMR and UV-vis explained in Quantification of ammonia of Materials and methods section. The white-grey color solid byproduct from the reaction residue is used to release and recover pure CO_2_. The directly lithium nitride hydrolysis experiment was conducted by loaded ~0.1 g of synthesised lithium nitride samples into a 50 mL syringe, then 5 mL of 4°C water (with 0.1 mL of 0.5% phenolphthalein solution per 50 mL) were added. The change of volume and colour were recorded as Video S1, S2 and Fig. 2f.

**CO_2_ release and yield**

5 mL of 2 M H_2_SO_4_ is added to the white-grey color solid products from the reaction between air and Li_3_N drop by drop using a syringe. During this step, the released CO_2_ is collected by accumulation of gases over PEG (polyethylene, saturated with CO_2_), then the mole of CO_2_ is calculated by the volume of gas and then can calculate the mass of Li_2_CO_3_ in the residue added. CO_2_ collected was extracted using a syringe and through microporous filter to remove water then introduced into the GC system (Agilent 7890B), equipped with a thermal conductivity detector (TCD), for analysis.

**Quantification of ammonia**

The synthesized ammonia in 0.05M H_2_SO_4_ is then quantified with two methods: (1) spectrophotometry using the indophenol blue method and (2) 1H nuclear magnetic resonance (1H NMR). In the spectrophotometry method, the following reagents were prepared:

1. Chromogenic solution: 2 g of NaOH was dissolved in 50mL of DI water, and 2.5 g of sodium salicylate (C_6_H_4_(OH)COONa), 2.5 g of potassium sodium tartrate (KNaC_4_H_6_O_6_·4H_2_O) was added.
2. Oxidizing solution: 3 mL of 5% NaClO is diluted by 33 mL of DI water.
3. Catalysing reagent: 0.225 g of sodium nitroprusside dihydrate, Na_2_[Fe(CN)_5_NO]·2H_2_O is dissolved in 20 mL of DI water.
4. Ammonia standard solution: 1000 μg NH_3_/mL solution is made by dissolving 0.3147 g of NH_4_Cl in 0.05 M H_2_SO_4_ and making volume of 100 mL, and the standard solution is diluted to a certain concentration to make standard curves.

Then 4 mL sample, 4 mL chromogenic solution, 2 mL oxidizing solution, and 0.4 mL catalysing reagent are mixed, well- shake and then stand still for 2 hours to achieve a stable absorbance. Then the sample is taken for a UV test of 500-800 nm using an UV-vis spectrophotometer. The same process was done several times for standard samples and standard curves.

The NMR tests were performed on Buker bio-600. The NMR samples were prepared by mixing 0.8mL of the sample solution or diluted sample solution use 0.05M H_2_SO_4_ with 0.2mL of d_6_-DMSO as solvent and internal standard. The standard samples were made in same way as the diluted ammonia standard solution. Pre-saturation for water suppression and 64 scans are used for all NMR measurements.

**Electrodeposit of lithium**

The deposition of lithium was carried out in a glass H-cell separated by LICGC film. A 1cm^2^ Pt plate was used as an anode and a 1cm^2^ Cu plate was served as a cathode during lithium deposition. The cathode electrolyte is 1 M LiClO_4_/PC and the anode electrolyte is 1M Li_2_SO_4_ aqueous solution. Then to test the performance, chronopotentiometry is carried out at a current density of 0.5-5 mA/cm^2^ several times for 10 minutes to 2 hours, and chronoamperometry has carried out at voltage from -4 V to -9.5 V for 10 minutes to 2 hours, using PalmSens4 electrochemical Station (Palmsens B.V.). The deposit of lithium was run inside a nitrogen atmosphere glovebox to prevent the oxygen and moisture when operating. After the reaction, the cathode is taken out from the solution, washed with THF, and dried inside the nitrogen glovebox (THF is highly volatile and easily to be removed).

**Li_3_N conversion rate quantification**

After the Li_3_N is made, measured amount of Li_3_N powders or plates were placed inside a flask and 0.05M H_2_SO_4_ was added into the flask to generate ammonia and the solutions were taken for UV-vis and 1H-NMR for ammonia quantification.

$${{Li}_{3}N}_{wt\%}=\frac{C_{{NH}_{3}}(mg {mL}^{-1})\times V_{H_{2}{SO}_{4}}(mL)\times M_{{Li}_{3}N}(g {mol}^{-1})}{M_{{NH}_{3}}(g {mol}^{-1})\times m(mg)}$$

Where C_NH3_ (mg/mL) was the concentration of ammonia, V_H2SO4_ was the volume of H_2_SO_4_ added into the flask, M_Li3N_ (g/mol) and M_NH3_ (g/mol) are the molar mass of lithium nitride and ammonia respectively, which is 34.83 and 17.03. m(mg) is the initial mass of lithium nitride samples.

**Faraday efficiency quantification**

The cathode material is then taken into a flask under N_2_ atmosphere with a continuous N_2_ flow of 60 L/min, and the system is heat to 80 °C for 12 hours for nitridation. Then 0.05 M H_2_SO_4_ was added into the flask to generate ammonia and the solutions were taken for UV-vis and ^1^H-NMR for ammonia quantification.

$${FE}_{{NH}_{3}}=\frac{C_{{NH}_{3}}(mg {mL}^{-1})\times V_{H_{2}{SO}_{4}}(mL)\times3\times F}{Q_{steady}(C)}$$

Where C_NH3_ (mg/mL) was the concentration of ammonia, V_H2SO4_ was the volume of H_2_SO_4_ added into the flask after N_2_ treatment, F was Faradaic constant (96485 C mol^-1^), and the Q_steady_ (C) was the total passed electrons during the steady state current in electrolysis. The steady state current was used for FE calculation (after the voltage stable shown in Figure S22) and initial SEI formation induced transient state are excluded since it’s not contributed to the formation of Li metal.

**Other characterizations**

The X-ray diffraction (XRD) patterns were acquired using Bruker D8 Advance instrument with Cu Kα radiation (λ = 1.5406 Å) at 40 mA and 40 kV with a scanning rate of 0.1° s^–1^. The composition of Li_3_N was calculated by the DIFFRAC.EVA V6.

The X-ray photoelectron spectroscopy (XPS) spectrums were obtained by a Thermo Scientific K-alpha system with Al Kα (1486.7 eV) radiation and calibrated by C 1s peak at 284.8 eV as the reference.

The transmission electron microscopy (TEM), high-resolution transmission electron microscopy (HR-TEM) were conducted on a JEOL JEM-F200 instrument. Before the TEM and HR-TEM test, the samples were suspended in ethanol, sonicated for 0.5 h, and then transferred to a nitrogen atmosphere and dropped onto a copper grid and dried before test.

The scanning electron microscopy (SEM) images were obtained by a Hitachi FlexSEM 1000 instrument.

The inductively coupled plasma-optical emission spectroscopy (ICP-OES) was determined by a Varian 720-ES ICP-OES.

**Density Functional Theory (DFT) calculations**

All the calculations are performed using the Vienna ab initio simulation package (VASP), which is based on the density functional theory^[1-2]^. The generalised gradient approximation implemented by Perdew, Burke, and Ernzerhof (PBE) pseudopotentials^[3]^ and the projector augmented wave (PAW) approach^[4]^ are employed. (ref) The plane wave basis cutoff is set as 400 eV. The energy convergence criterion is set to 10^-5^ eV, and the residual forces in the converged structures are smaller than 0.01 eV/Å.

We construct supercell slabs for α-Li_3_N and β-Li_3_N with different orientations and numbers of atomic layers. For the α-Li_3_N (001), we create a 2×2 supercell slab with six layers of atoms, fixing the bottom three layers. α-Li_3_N (001) supercell slab with eight layers is also constructed, where the bottom four layers are fixed. For the β-Li_3_N, we prepare three different orientations: a 2×2 (100) supercell, a 4×2 (100) supercell slab, and a 4×2 (101) supercell slab, both with nine layers of atoms, and in each case, the bottom five layers are fixed. The Brillouin zone is sampled by a gamma-center 3×3×1 grid. The vacuum space is larger than 15 Å to minimize the spurious interactions between the periodically repeated images. The weak dispersive force is described by the DFT-D3 semi-empirical method.^[5]^

The surface energy in a function of nitrogen is defined as

$$\gamma= \frac{1}{2A} (E_{slab} - \frac{1}{3}n_{Li}E_{bulk} - \frac{u_{N}}{3}(n_{Li}-3n_{N}))$$

where *A* is the area of Li_3_N surface, *E*_slab_ is the total energy of Li_3_N slab, *E*_bulk_ is the energy of Li_3_N bulk, *n*_N_ and *n*_Li_ are the number of N and Li atoms in the Li_3_N slab, and the *u*_N_ is half of the chemical potential of nitrogen.

The adsorption energy of H_2_O molecule is defined as

$$E_{ads}=E_{tot}-E_{surf}-E_{molecule}$$

where *E*_tot_ is the total energy of H_2_O molecule adsorbed on Li_3_N surface, *E*_surf_ is the total energy of Li_3_N surface, and *E*_molecule_ is the energy of a H_2_O molecule in vacuum.

**Supplementary Notes**

**Supplementary Notes 1:**

The electrochemical lithium-mediated nitrogen reduction reaction (Li-NRR) in organic electrolyte systems has been extensively studied by MacFarlane, Nørskov, and their respective groups^[6-17]^. In most cases, **their work employs strategies such as using alcohols as sacrificial proton carriers or introducing hydrogen oxidation reactions (gaseous hydrogen) at the anode** to supply protons for ammonia (NH_3_) formation. Specifically, in MacFarlane's work, the use of trihexyltetradecylphosphonium ([P_6,6,6,14_]⁺) as a robust proton shuttle has been identified as a key component for facilitating lithium mediation. Meanwhile, Nørskov's work often involves **high-pressure N_2_** or flow reactors with continuous N_2_ supply to achieve high efficiency in NH_3_ production. We have carefully cited their pioneering contributions and acknowledge that these groups are at the forefront of developing green ammonia synthesis technologies.

**Supplementary Notes 2:**

In Figure 2a, the x axis represents the detailed different pre-activation time for the lithium plate under consistent nitridation conditions, where the precursors after pre-activation are consequently exposed to N_2_ for 24 hours at 80°C. The y axis shows the total Li_3_N generated in each sample. This figure indicates that there is an optimized pre-activation time for this stage.

In Figure 2b and 2c, the X axis represents different pre-activation times. After the pre-activation, the precursors are taken for N_2_ treatment and generate Li_3_N. However the y axis differs between two figures, Figure 2b corresponds to N_2_ treatment for 3 hours, while Figure 2c corresponds to N_2_ treatment for 24 hours. By the comparison of 2b and 2c we acknowledge that the optimized pre-activation time is around 12 hours. When at shorter pre-activation times such as 4 hours, the activation is incomplete, the kinetics is slow as the conversion rate increases with longer N_2_ treatment time. However, at an extended pre-activation time such as 20 hours, the conversion rate keeps almost same low for different nitridation time, which indicates that the under excessive activation, the form of Li_3_N is rapidly but too much moisture is reacted and consumed the Li plate during pre-activation, leads to the unchanged low conversion rate.

Figure 2f illustrates the exothermic hydrolysis of Li_3_N, with details provided in Video S1. In this video, 0.12 g of as-synthesised Li_3_N was loaded into a 50 mL syringe, followed by 5 mL of deionized water (80 equivalents, 4°C, phenolphthalein as pH indicator). As the Li_3_N hydrolysis reaction progressed, gaseous and partially dissolved NH_3_ was generated, causing the syringe volume to expand to approximately 25 mL. Upon the complete consumption of Li_3_N, the syringe naturally returns to room temperature, the NH_3_ gradually dissolved in H_2_O, leading to a reduction in syringe volume. The red color represents the formation of alkaline species, primarily LiOH and NH_3_·H_2_O. Notably, at higher alkaline concentrations, the red color becomes lighter, consistent with the observed results.

Video S2 is the control experiment for Video S1, conducted without added pH indicator and the water is at room temperature. In this case, the absence of low-temperature water to control the reaction rate resulted in a rapid expansion of the syringe volume, reaching up to 60 mL. However, after the syringe naturally return to room temperature, the syringe volume is similar to that observed in Video S1.

**Supplementary Notes 3:**

The Li_3_N synthesis rates was based on the time for pre-activation and nitridation, and the product is Li Li_3_N.

$$r_{Li_{3}N}= \frac{1}{M_{Li}(g)}\frac{N_{Li_{3}N}(mmol)}{t_{pa}(hr)+t_{n}(hr)}$$

Where M_Li_ (g) is the mass of lithium, N_Li3N_ (mmol) was the molar of Li_3_N produced, t_pa_ (hr) was the pre-activation time and t_n_ (hr) was the nitridation time.

The NH_3_ release rate was calculated based on the time for introducing air flow, the amount of Li_3_N, and the amount of NH_3_ released.

$$r_{{NH}_{3} release}= \frac{1}{M_{Li_{3}N}}\frac{N_{{NH}_{3}}}{t_{air}}$$

Where M_Li3N_ (g) is the mass of lithium nitride, N_NH3_ (mmol) was the molar of NH_3_ produced, t_pa_ (hr) was the air treating time.

The DAC rate was calculated based on the time for introducing air flow, the amount of CO_2_ absorbed, and the amount of absorbents, which identify as Li_2_CO_3_ here.

$$r_{DAC}= \frac{1}{M_{LiOH}}\frac{N_{CO_{2}}}{t_{air}}$$

Where M_LiOH_ (g) is the mass of lithium hydroxide (based on the mass of lithium nitride), N_CO2_ (mmol) was the molar of CO_2_ absorbed, t_pa_ (hr) was the air treating time.

**Supplementary Notes 4:**

Typically, commercial plants with traditional Haber-Bosch process consumes 46 GJ energy per ton of NH_3_^[18]^, with the releasing of 2.4 ton of CO_2_ in average^[19]^. These energy sources are mainly sourced from fossil fuel and are challenging to be replaced by renewable energy. The typical DAC process also requires energy for the regeneration of adsorbents or absorbents (e.g. by heating up to decompose and release CO_2_ or pressure swing to desorption), and the average energy consumption of DAC is ~10 GJ per ton of CO_2_ (target values specified by the respective companies)^[20]^.

In our devised process, since all the reaction steps except Li recycling are spontaneous and performed at or near room temperature, the energy consumption of ~147 GJ /t NH_3_ can be achieved with 3.4 t of CO_2_ captured from air (thro. Max. capture 3.84 t of CO_2_), which on average can potentially save 34 GJ for capture CO_2_ from air and results in ~113 GJ /t NH_3_.

The theoretical energy cost can be calculated based on the electrochemical step:

$$W=\frac{U\times Q_{total}}{FE}$$

Where U is the required voltage at maximum efficiency, Q_total_ is the total passed electrons for 1 ton NH_3_, FE is the Faraday efficiency. And the calculated optimized gross energy consumption is ~64.7 GJ /t NH_3_, the net optimized energy consumption considering DAC energy saving is ~23.6 GJ /t NH_3_.

$$Energy cost=Energy-Energy saved by DAC$$

$$64.7 GJ-3.84 ton {CO}_{2}\times10{GJ}/{ton {CO}_{2}}=26.3GJ$$

**Supplementary Tables**

**Table S1**: The Bader charge analysis of Li(1) and Li(2) on α/β-Li_3_N bulk and surfaces.

| Electron loss (*e*) | bulk | | α-Li_3_N surface | | β-Li_3_N surface | | |
| --- | --- | --- | --- | --- | --- | --- | --- |
|  | α-Li_3_N | β-Li_3_N | (001) | (100) | (001) | (100) | (101) |
| Li(1) | 0.82 | 0.82 | 0.74 | 0.83 | 0.84 | 0.85 | 0.81 |
| Li(2) | 0.82 | 0.82 | 0.74 | 0.85 | 0.81 | 0.82 | 0.85 |

**Table S2**: The comparison of CO_2_ absorption performance.

| Name | CO_2_ uptake (mmolCO_2_/g absorbents) | CO_2_ fraction v/v | Category | Ref |
| --- | --- | --- | --- | --- |
| Mg-MOF-74 | 3.9 | 400 ppm | MOFs | ^[21]^ |
| MgO-RHA-20 | 4.56 | 0.1 |  | ^[22]^ |
| SH800 | 6.77 | 0.5 |  | ^[23]^ |
| FBNNSs/ZnO | 2.83 | 1 |  | ^[24]^ |
| [Mg_2_(dobdc)(N_2_H_4_)_1.8_] | 4.2 | 0.15 |  | ^[25]^ |
| AC-KOH-N | 7.19 | 1 |  | ^[26]^ |
| MEA(30-40 wt%) | 9.18 | 400 ppm | aqueous amine/amino acids | ^[27]^ |
| glyk | 2.03 | 400 ppm |  | ^[28]^ |
| glyk-m-BBIG | 5.66 | 400 ppm |  | ^[29]^ |
| Taurine | 8.79 | 1 |  | ^[30]^ |
| NaOH | 11.75 | 380ppm | aqueous alkaline | ^[31]^ |
| Ca(OH)_2_ | 13.5 | 380ppm |  | ^[31]^ |
| PZ-K_2_CO_3_ | 7.6 | 0.12 |  | ^[32]^ |
| DEAE | 5.2 | 0.2 |  | ^[33]^ |
| 1M-2PPE | 5.19 | 0.2 |  | ^[33]^ |
| 1 g-F-Ca(OH)_2_ | 69 | 0.3 |  | ^[34]^ |
| Zn(Ac)_2_/PIL-Ac | 2.5 | 1 | ionic liquids | ^[35]^ |
| MDEA/[Omin][BF_4_] | 11.09 | 1 |  | ^[36]^ |
| Resin KBS-PEI | 1.59 | 4000 ppm | aminopolymer-solid | ^[37]^ |
| Resin MR10-PEI | 2.92 | 1000 ppm |  | ^[38]^ |
| Resin HP2MGL-PEI | 3.04 | 5000 ppm |  | ^[39]^ |
| Resin HP2MGL-PEI | 1.96 | 400 ppm |  | ^[39]^ |
| MCF pellets-PEI | 2.52 | 420 ppm |  | ^[40]^ |
| Hierarchical silica-PEI | 3.36 | 400 ppm |  | ^[41]^ |
| PEI–Mg_0.55_Al–O | 2.27 | 400 ppm |  | ^[42]^ |
| TEPA–Mg_0.55_Al–O | 3 | 400 ppm |  | ^[43]^ |
| E-VER-TEPA-2% | 29.5 | 1 |  | ^[44]^ |
| KH540-NS–NR2 | 25 | 1 |  | ^[45]^ |
| TEPA/SBA-15 | 3.4 | 400 ppm |  | ^[46]^ |
| PEI/SBA-15 | 1.12 | 400 ppm |  | ^[46]^ |
| PEI@AOMC | 2.58 | 1 |  | ^[47]^ |

**Table S3**: The comparison of ammonia production rate under ambient conditions.

| H element source | key chemicals | rate | ref |
| --- | --- | --- | --- |
| **Directly air** | Li | 4.5 µmol h^–1^ cm^-2^ | This work |
| Water | Sn_SC_/C | 1.02 µmol h^–1^ mg^–1^ | ^[48]^ |
|  | Bi@C | 0.25 µmol h^–1^ mg^–1^ | ^[49]^ |
|  | Fe-doped TiO_2_ | 1.50 µmol h^–1^ mg^–1^ | ^[50]^ |
|  | FeWSx@FeWO_4_ | 1.78 µmol h^–1^ mg^–1^ | ^[51]^ |
|  | Nanoporous Pd_3_Bi | 3.47 µmol h^–1^ mg^–1^ | ^[52]^ |
|  | Li-TiO_2_ nanosheets | 0.51 µmol h^–1^ mg^–1^ | ^[53]^ |
|  | mAu_3_Rh/NF | 1.55 µmol h^–1^ mg^–1^ | ^[54]^ |
|  | Ag nanosheet | 0.17 µmol h^–1^ cm^-2^ | ^[55]^ |
| Ethanol | [P_6,6,6,14_][eFAP] | 190.8 µmol h^–1^ cm^-2^ | ^[7]^ |
|  | [P_6,6,6,14_][eFAP], LiNTf_2_ | 540 µmol h^–1^ cm^-2^ | ^[9]^ |
| Hydrogen gas | Ca[B(hfip)_4_]_2_/Ca(BH4)2 | not reported | ^[11]^ |
|  | LiBF_4_, PtAu | not reported | ^[12]^ |

**Table S4:** The bond length analysis of H_2_O decomposition on α/β-Li_3_N surfaces.

| Bond length (Å) | α-Li_3_N surface | | β-Li_3_N surface | | |
| --- | --- | --- | --- | --- | --- |
|  | (001) | (100) | (001) | (100) | (101) |
| Li-O | 1.85 | 1.80 | 1.93 | 1.79 | 1.86 |
| N-H | 1.79 | 1.03 | 1.04 | 1.03 | 1.04 |

Interestingly, Li-O and N-H bonds formed upon H_2_O decomposition on these four surfaces. From the simulation results above, the Li-O bond length ranges from ~1.8 to 1.9 Å, while the N-H bond length is approximately 1.03 Å, consistent with the bond lengths of LiOH (~1.85Å)^[56]^ and NH_3_ (~1.01Å)^[57]^. This further confirms that the reaction of H_2_O and Li_3_N surfaces leads to the formation of LiOH and NH_3_.

**Supplementary Figures**


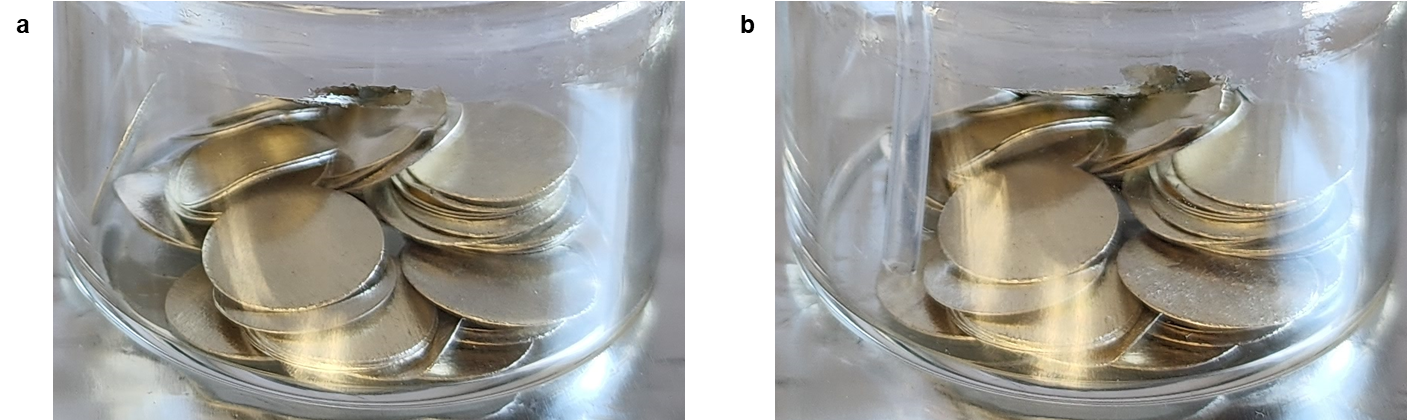


**Figure S1**: Control experiments showing negligible Li surface reaction without pre-activation step. **a**. the lithium plates stored under dry nitrogen for 2 months. **b**. the lithium plates with 2 hours exposure to continuous flow of N_2_ and 80 °C.


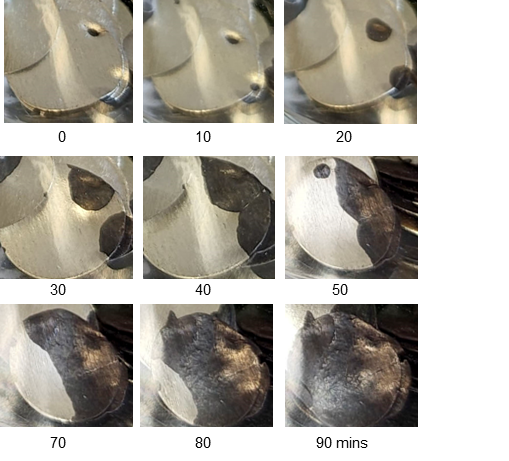


**Figure S2**: Sequential photos of a Li plate during N_2_ fixation. Prior to imaging the sequence, Li plate was exposed to air for duration of 5 s in pre-activation step proof of concept. Photos show growth of Li_3_N grains in dark color.


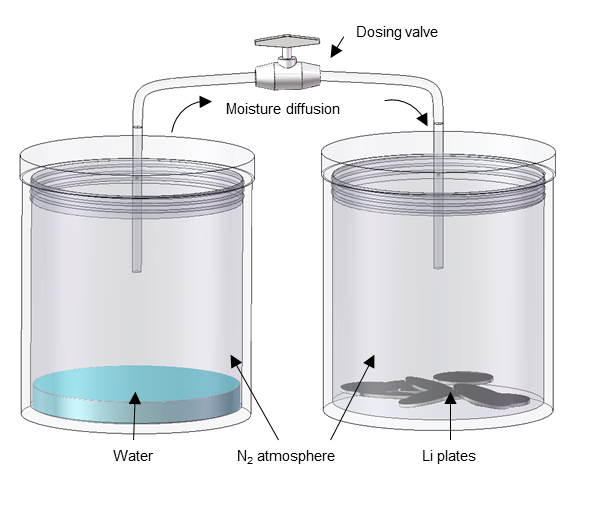


**Figure S3**: Schematic of activation step including controlled exposure of Li plate to moisture prior to nitridation reaction.


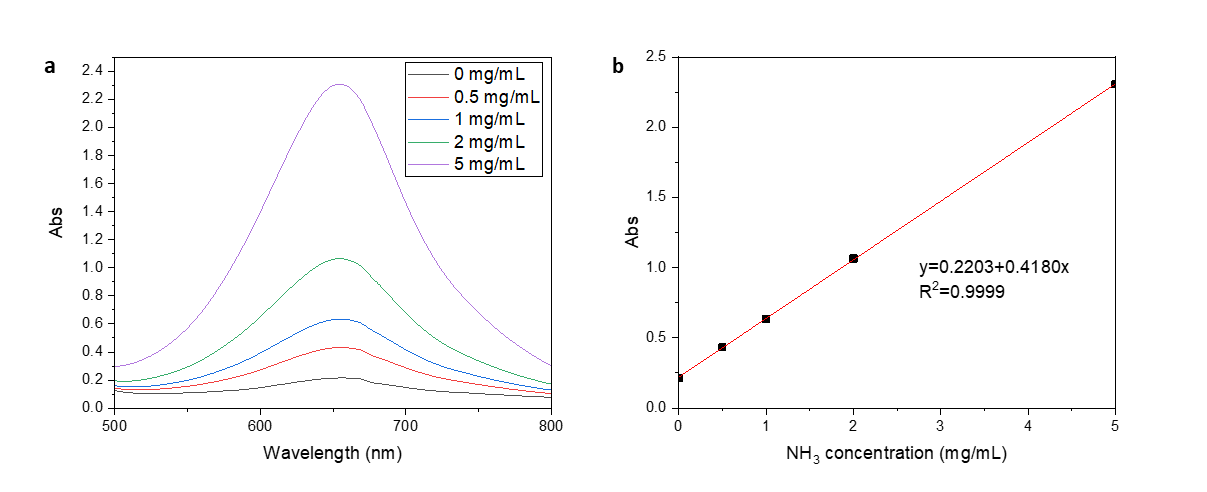


**Figure S4**: **a**. UV-Vis absorption spectra of indophenol blue method measured from NH_3_ standard solutions. **b**. calibration curve for indophenol blue method at 655 nm used in this study. To quantify the concentration of ammonia, UV-Vis and ^1^H-NMR is utilized throughout the study. Figure S4 and 5 display the UV-Vis spectra and the corresponding calibration curves for the NH_3_ standard solutions.


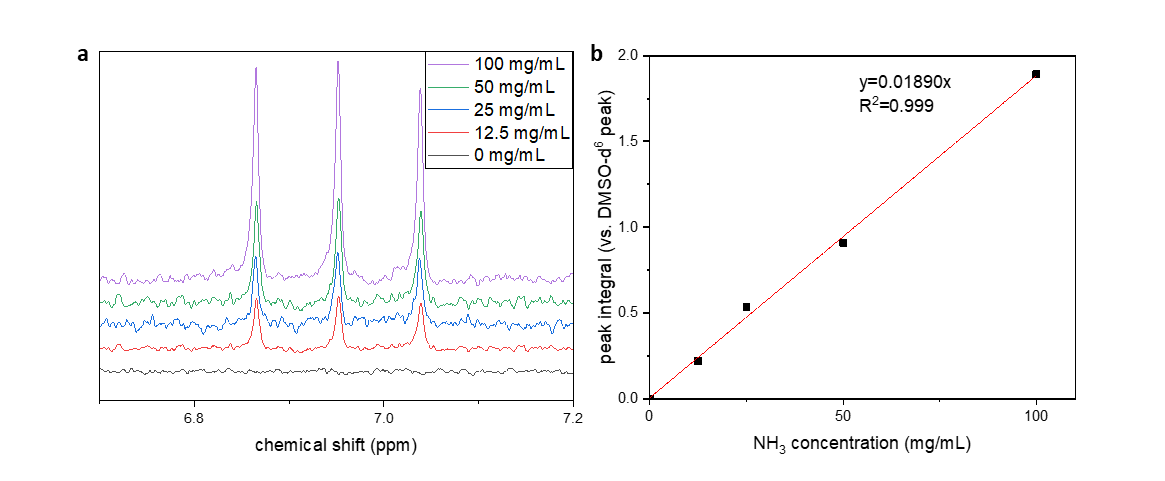


**Figure S5**: **a**. ^1^H-NMR spectrum of the standard solution with different concentrations of NH_3_. **b.** calibration curve for ^1^H-NMR method used in this study.


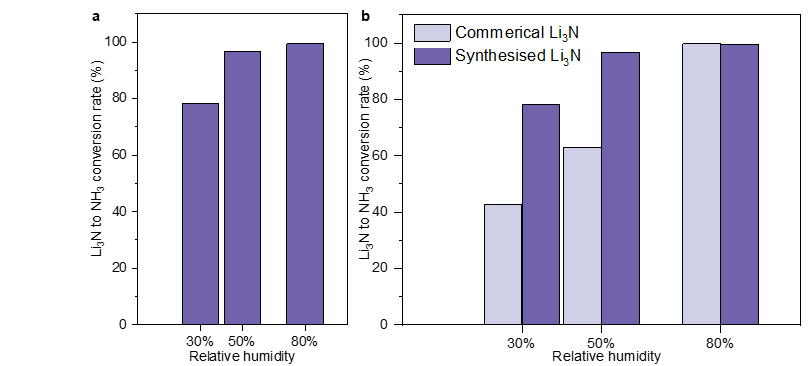


**Figure S6**: **a**. Conversion efficiency of synthesised (bmss) Li_3_N to ammonia under 2.5 L/min air flow and different relative humidities with the duration of 24 hrs. **b**. Comparison of conversion efficiency of synthesised (bmss) Li_3_N and commercial Li_3_N (Fig 2d).


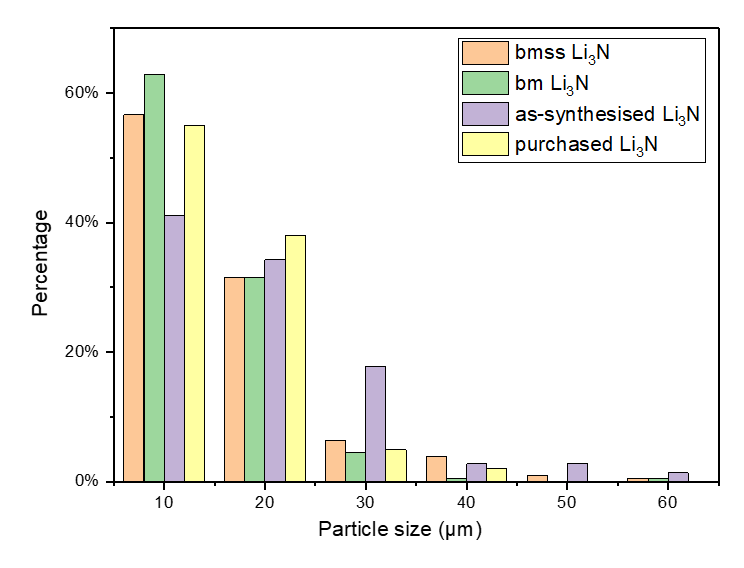


**Figure S7**: The particle size distribution of bm Li_3_N, bmss Li_3_N, as-synthesised Li_3_N and purchased Li_3_N


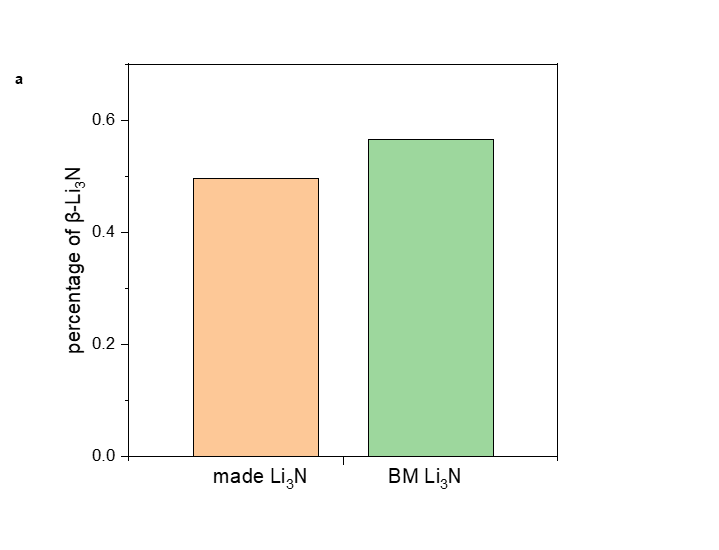


**Figure S8**: the TEM counts percentage for β-Li_3_N


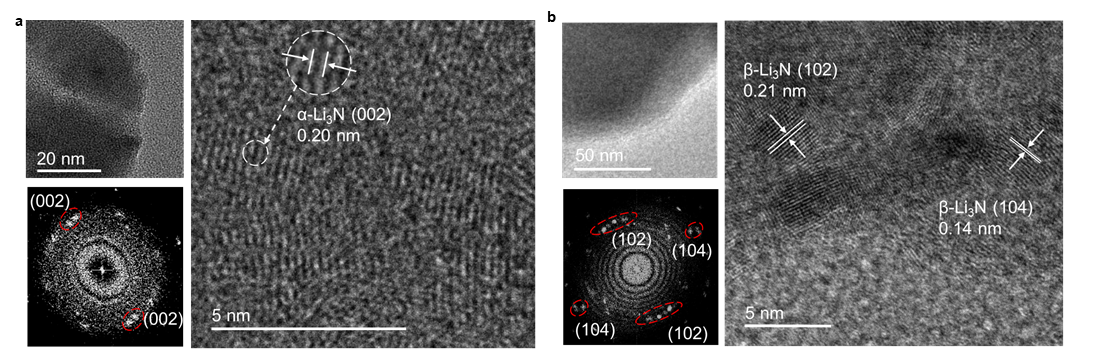


**Figure S9**: Structural characterisation of Li_3_N as-synthesised and bmss samples. HRTEM images of **a**. as-synthesised Li_3_N, and **b**. bmss, with the inset showing the fast Fourier transform (FFT) of the micrograph with the indexed crystal planes.


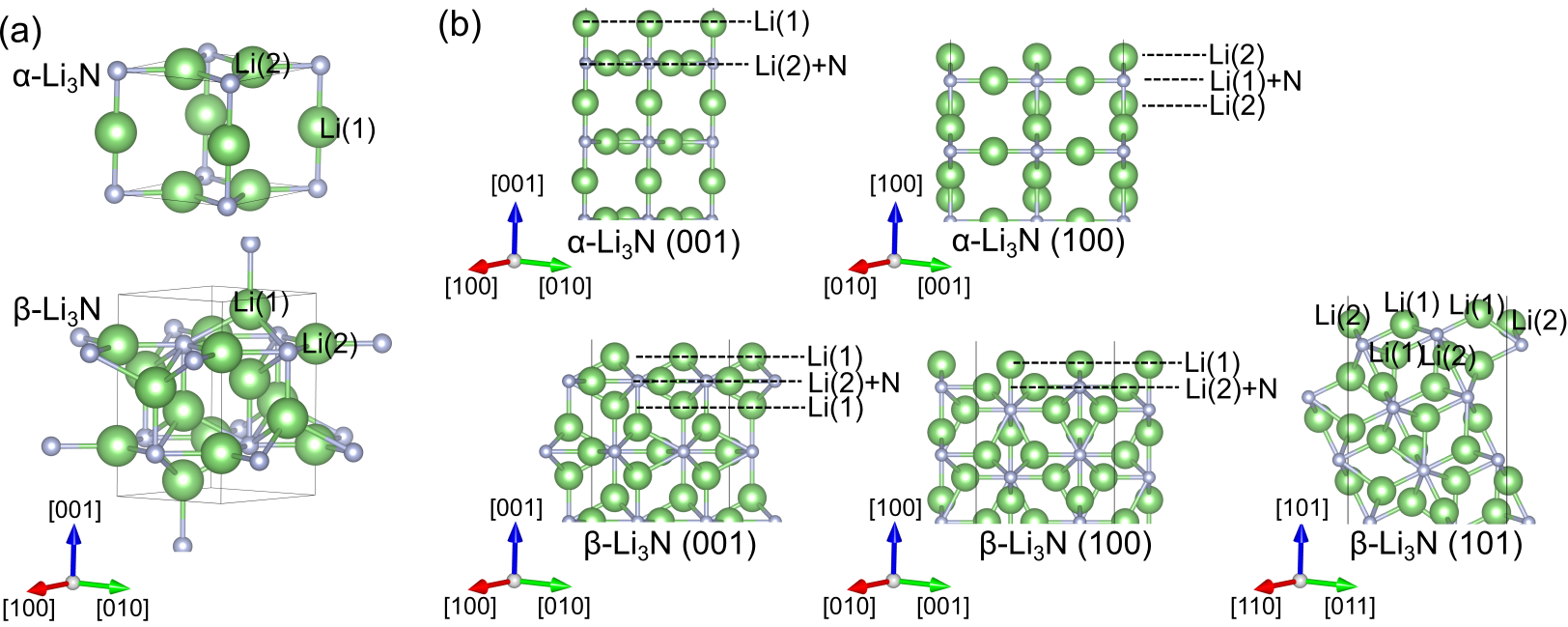


**Figure S10**: **a**. The crystal structures of bulk α/β-Li_3_N **b**. Schematic of α/β-Li_3_N surfaces as identified by XRD results with different terminated atoms.

**
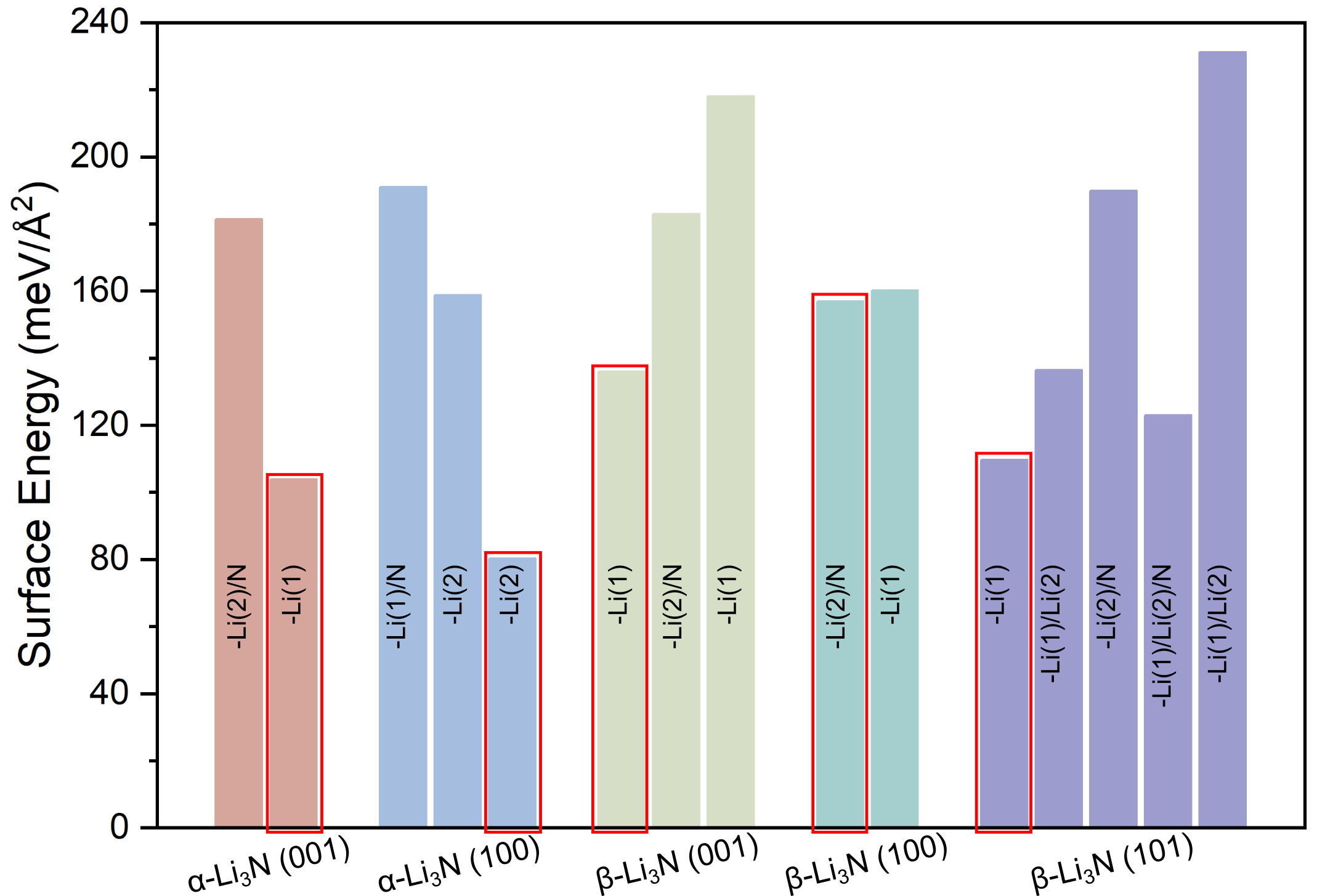
**

**Figure S11**: Calculated surface energy (γ) of various α/β-Li_3_N surfaces under 1 atm pressure at 353.15 K. Red rectangles marked the most energetically favourable surfaces for each surface orientation.

**
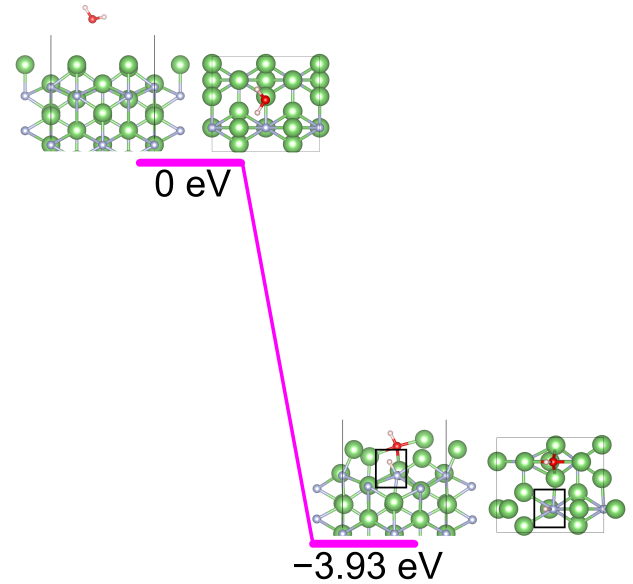
**

**Figure S12**: The top and side views of the adsorption and decomposition of H_2_O on a β-Li_3_N surface terminated with a Li(1) atom. The top view includes only the top four layers.


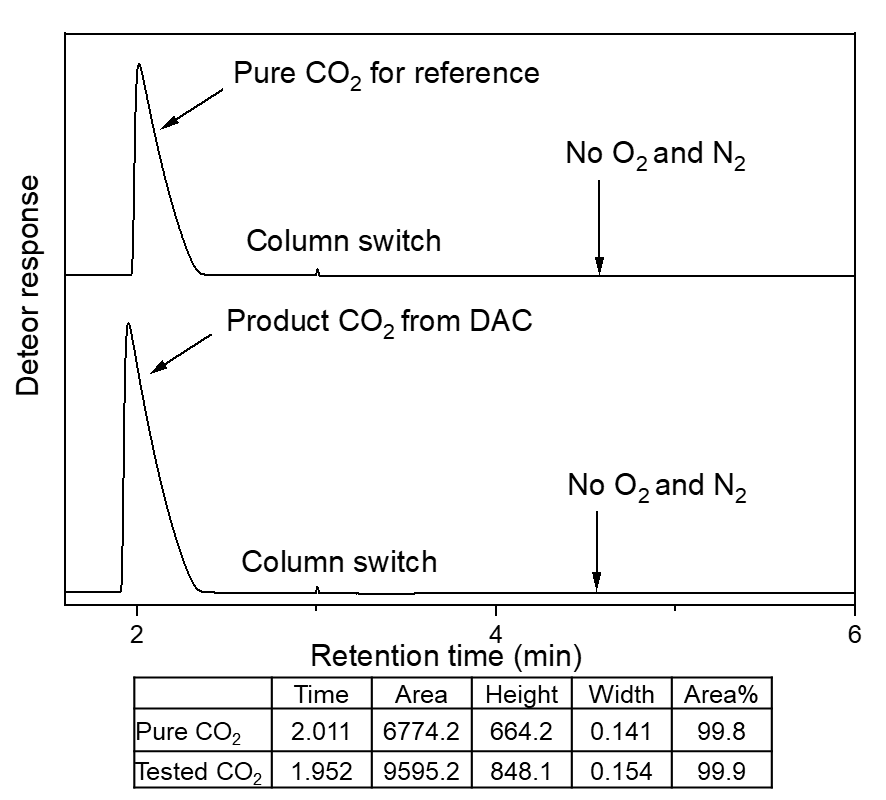


**Figure S13**: The GC figure for pure CO_2_ release and the GC figure for the collected gas after acid is added to Li_2_CO_3_.


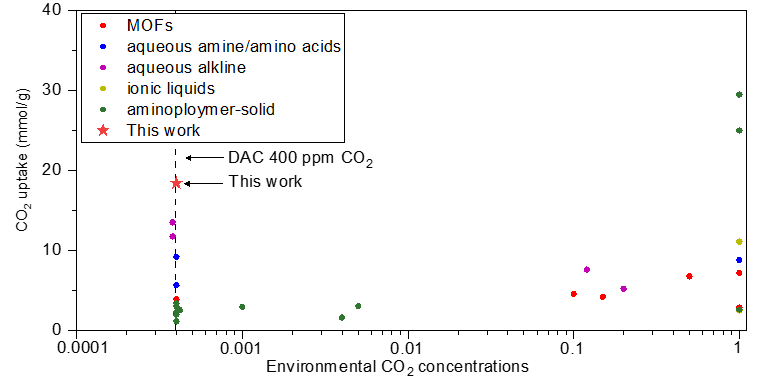


**Figure S14**: The comparison of DAC CO_2_ uptake in this work and several other CO_2_ absorbing agents ^[22, 24-27, 29, 31-33, 36, 44-47, 58]^.


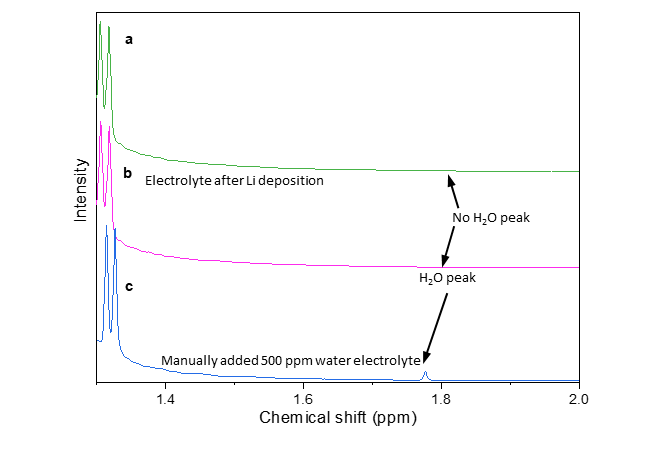


**Figure S15**: **a**, ^1^H-NMR of cathode electrolyte before experiment and **b**, after 2 days of lithium deposition, **c**, electrolyte with 1 droplet of water for reference, 0.5 mL CDCl_3_ is added to 0.5 mL of samples for NMR test, the water peak present at 1.78 ppm.


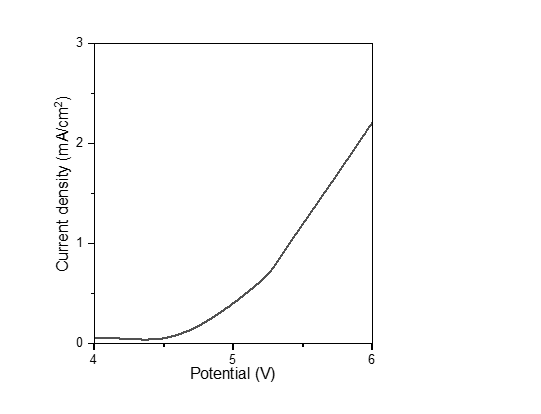


**Figure S16**: The Linear Sweep Voltammetry (LSV) figure for the LICGC cell. (running at 25 °C, cathode: Cu, anode: Pt, cathode electrolyte: 1M LiClO_4_/PC, anode electrolyte: 1 M Li_2_SO_4_, cathode area 4 cm^2^).


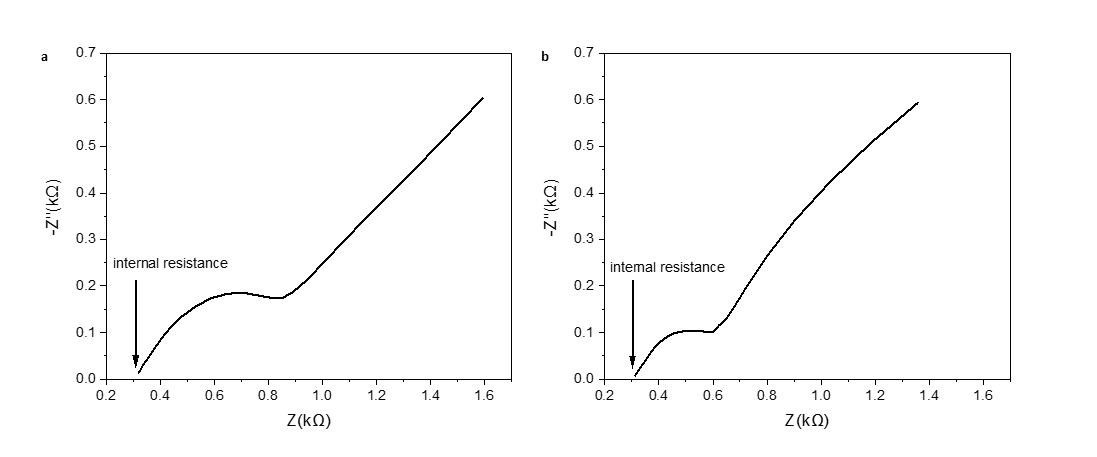


**Figure S17**: **a**, The EIS figure of new LICGC membrane and **b,** membrane under 10 hours test.


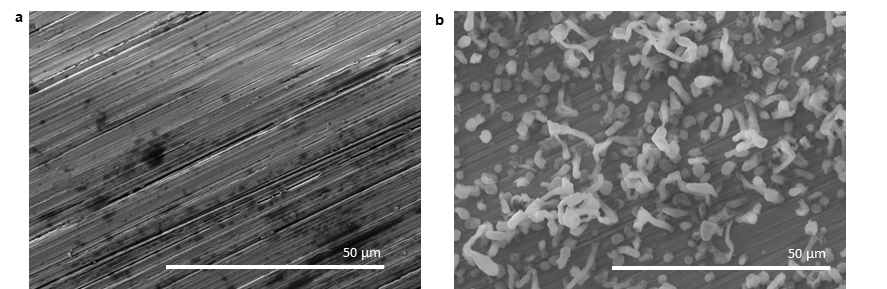


**Figure S18:** The SEM image of the cathode electrode surface after 1 hour, with current densities of **a**, 0.5 mA/cm^2^ and **b**, before electrolysis, respectively. The experiment was performed at 25 °C. Cathode and anode used were Cu and Pt, respectively with cathode area of 1 cm^2^. The cathode electrolyte included 1M LiClO_4_/PC and the anode electrolyte consisted of 1 M Li_2_SO_4_.


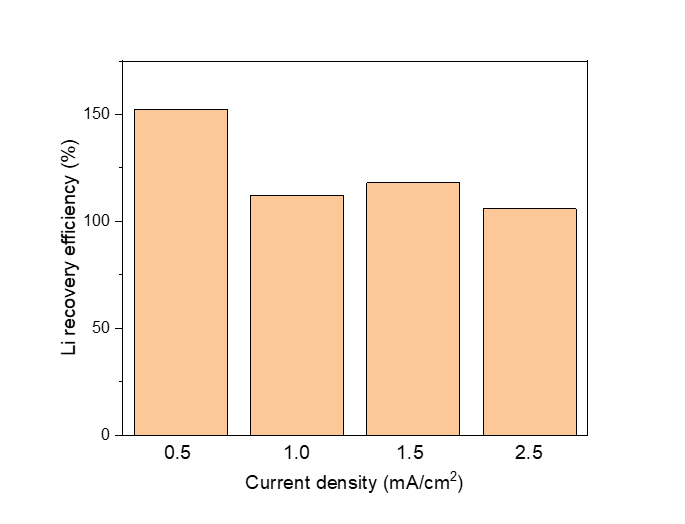


**Figure S19**: The Li recovery efficiency with different current density, generated by ICP-OES, calculated by the total Li^+^ species on cathode and the theoretical Li concentration if the FE is 100%.


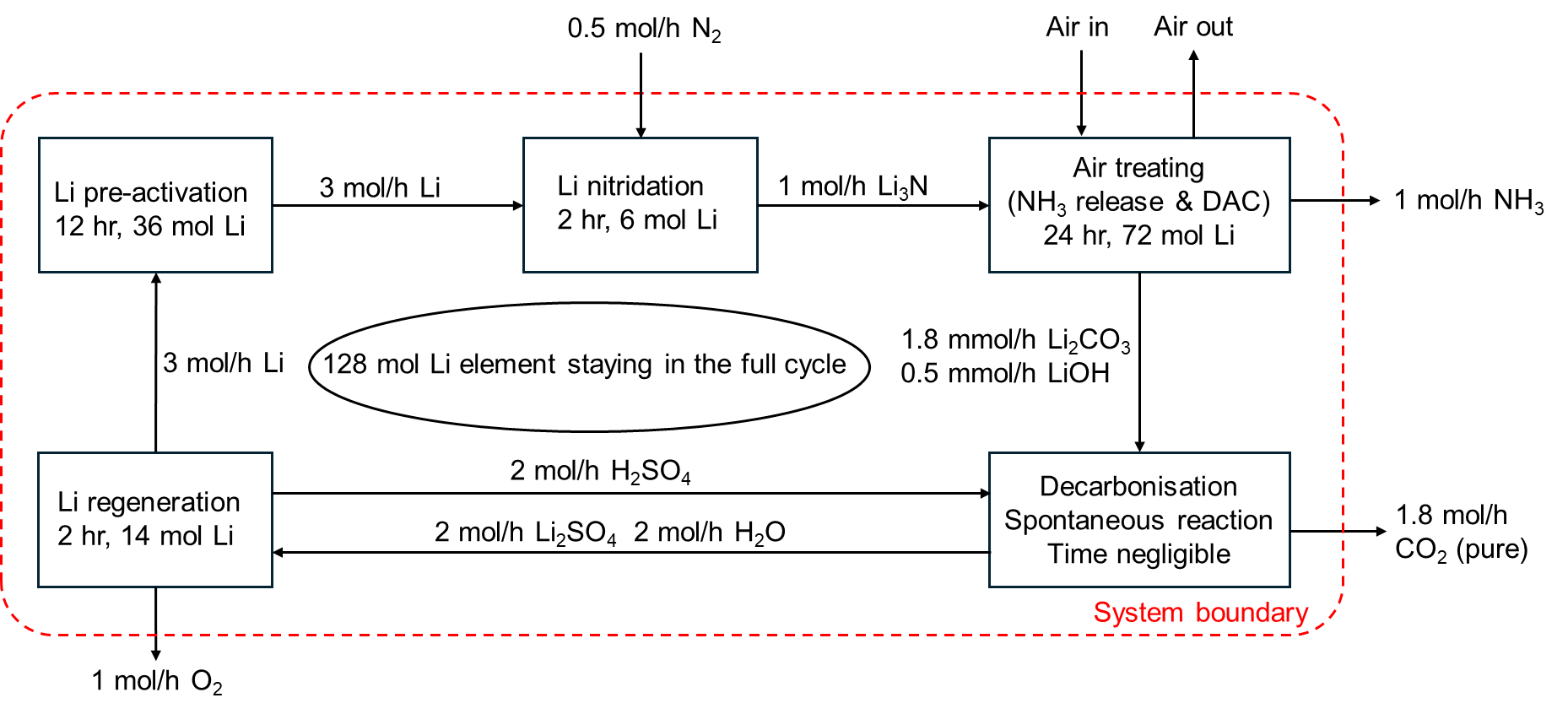


**Figure S20**: Process flow diagram of the Li-assisted chemical looping system for DAC and NH_3_ synthesis, with the molar amount of lithium shown in each block indicating the quantity of Li element stay in that step.


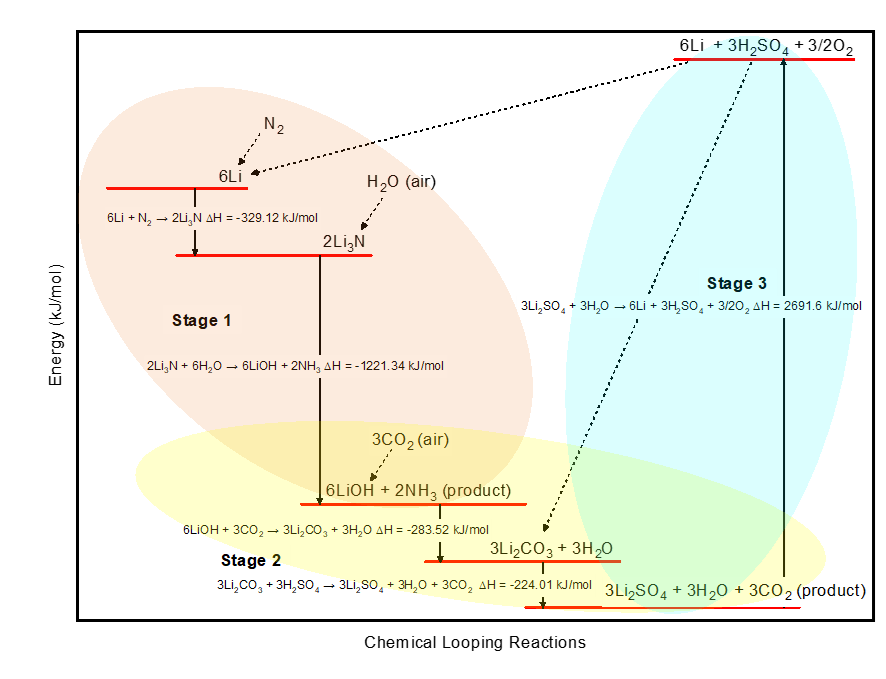


**Figure S21**: Detailed energy profiles of the three stage Li-assisted chemical loop, with equations specificized.


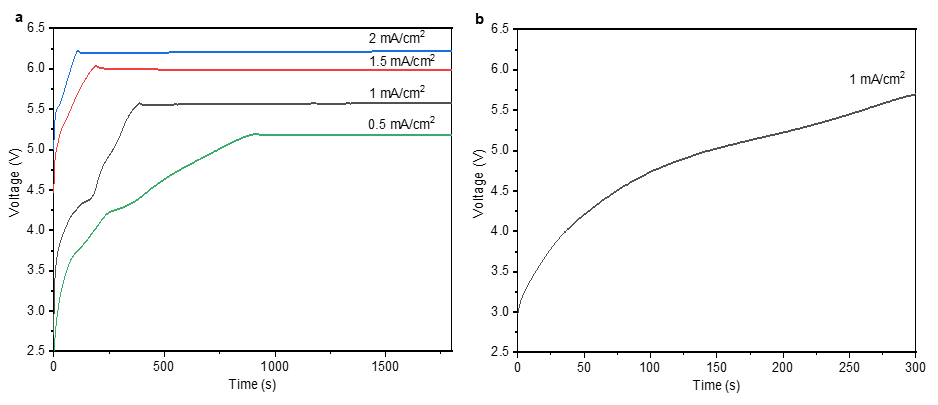


**Figure S22**: **a**, Voltage-time curve for the Li electrodeposition with different current density for 1800s. **b**, Voltage-time curve for the Li electrodeposition before steady state current achieved, at 1mA/cm^2^ for 300s, No or negligible FE is detected in the experiment. All experiments were performed at 25 °C. Cathode and anode used were Cu and Pt, respectively with cathode area of 1 cm^2^. The cathode electrolyte included 1M LiClO_4_/PC and the anode electrolyte consisted of 1 M Li_2_SO_4_.


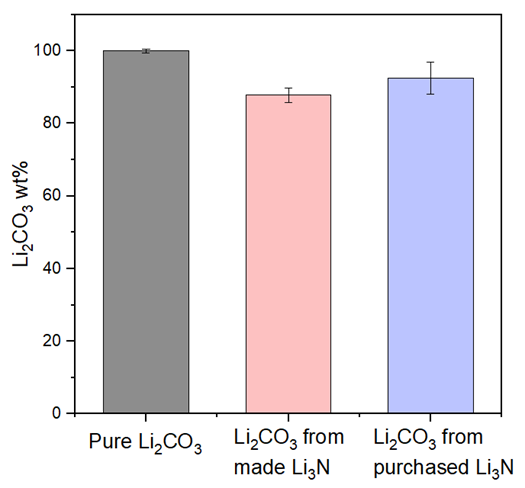


**Figure S23**: Weight percentage of Li_2_CO_3_ after the reaction between Li_3_N and air, measured based on the volume of CO_2_ released.

**Reference**

[1] G. Kresse, J. Furthmüller, *Physical Review B* **1996**, *54*, 11169-11186.

[2] G. Kresse, J. Furthmüller, *Computational Materials Science* **1996**, *6*, 15-50.

[3] J. P. Perdew, K. Burke, M. Ernzerhof, *Physical Review Letters* **1996**, *77*, 3865-3868.

[4] P. E. Blöchl, *Physical Review B* **1994**, *50*, 17953-17979.

[5] S. Grimme, J. Antony, S. Ehrlich, H. Krieg, *The Journal of Chemical Physics* **2010**, *132*, 154104.

[6] M. Krebsz, R. Y. Hodgetts, S. Johnston, C. K. Nguyen, Y. Hora, D. R. MacFarlane, A. N. Simonov, *Energy & Environmental Science* **2024**, *17*, 4481-4487.

[7] B. H. R. Suryanto, K. Matuszek, J. Choi, R. Y. Hodgetts, H.-L. Du, J. M. Bakker, C. S. M. Kang, P. V. Cherepanov, A. N. Simonov, D. R. MacFarlane, *Science* **2021**, *372*, 1187-1191.

[8] N.-T. Nguyen, L. A. O’Dell, K. N. Dinh, R. Y. Hodgetts, C. K. Nguyen, K. Banerjee, D. T. H. Truong, J. M. Bakker, A. McKay, D. R. MacFarlane, H.-L. Du, A. N. Simonov, *Chem* **2024**, *10*, 3622-3633.

[9] H.-L. Du, M. Chatti, R. Y. Hodgetts, P. V. Cherepanov, C. K. Nguyen, K. Matuszek, D. R. MacFarlane, A. N. Simonov, *Nature* **2022**, *609*, 722-727.

[10] R. Y. Hodgetts, H.-L. Du, T. D. Nguyen, D. MacFarlane, A. N. Simonov, *ACS Catalysis* **2022**, *12*, 5231-5246.

[11] X. Fu, V. A. Niemann, Y. Zhou, S. Li, K. Zhang, J. B. Pedersen, M. Saccoccio, S. Z. Andersen, K. Enemark-Rasmussen, P. Benedek, A. Xu, N. H. Deissler, J. B. V. Mygind, A. C. Nielander, J. Kibsgaard, P. C. K. Vesborg, J. K. Nørskov, T. F. Jaramillo, I. Chorkendorff, *Nature Materials* **2024**, *23*, 101-107.

[12] X. Fu, A. Xu, J. B. Pedersen, S. Li, R. Sažinas, Y. Zhou, S. Z. Andersen, M. Saccoccio, N. H. Deissler, J. B. V. Mygind, J. Kibsgaard, P. C. K. Vesborg, J. K. Nørskov, I. Chorkendorff, *Nature Communications* **2024**, *15*, 2417.

[13] S. Li, Y. Zhou, X. Fu, J. B. Pedersen, M. Saccoccio, S. Z. Andersen, K. Enemark-Rasmussen, P. J. Kempen, C. D. Damsgaard, A. Xu, R. Sažinas, J. B. V. Mygind, N. H. Deissler, J. Kibsgaard, P. C. K. Vesborg, J. K. Nørskov, I. Chorkendorff, *Nature* **2024**, *629*, 92-97.

[14] X. Fu, J. B. Pedersen, Y. Zhou, M. Saccoccio, S. Li, R. Sažinas, K. Li, S. Z. Andersen, A. Xu, N. H. Deissler, J. B. V. Mygind, C. Wei, J. Kibsgaard, P. C. K. Vesborg, J. K. Nørskov, I. Chorkendorff, *Science* **2023**, *379*, 707-712.

[15] S. Li, Y. Zhou, K. Li, M. Saccoccio, R. Sažinas, S. Z. Andersen, J. B. Pedersen, X. Fu, V. Shadravan, D. Chakraborty, J. Kibsgaard, P. C. K. Vesborg, J. K. Nørskov, I. Chorkendorff, *Joule* **2022**, *6*, 2083-2101.

[16] K. Li, S. Z. Andersen, M. J. Statt, M. Saccoccio, V. J. Bukas, K. Krempl, R. Sažinas, J. B. Pedersen, V. Shadravan, Y. Zhou, D. Chakraborty, J. Kibsgaard, P. C. K. Vesborg, J. K. Nørskov, I. Chorkendorff, *Science* **2021**, *374*, 1593-1597.

[17] J. M. McEnaney, A. R. Singh, J. A. Schwalbe, J. Kibsgaard, J. C. Lin, M. Cargnello, T. F. Jaramillo, J. K. Nørskov, *Energy & Environmental Science* **2017**, *10*, 1621-1630.

[18] J. Allen, S. Panquet, A. Bastiani, *Frontiers in Chemical Engineering* **2021**, *3*.

[19] IEA, IEA, **2021**.

[20] P. Viebahn, A. Scholz, O. Zelt, *Energies* **2019**, *12*, 3443.

[21] Y. Miao, Z. He, X. Zhu, D. Izikowitz, J. Li, *Chemical Engineering Journal* **2021**, *426*, 131875.

[22] Y. Guo, C. Tan, J. Sun, W. Li, J. Zhang, C. Zhao, *Fuel* **2020**, *259*, 116298.

[23] S. A. Anuar, W. N. R. Wan Isahak, M. S. Masdar, *International Journal of Energy Research* **2020**, *44*, 3148-3159.

[24] C. Yang, D. Liu, Y. Chen, C. Chen, J. Wang, Y. Fan, S. Huang, W. Lei, *ACS Applied Materials & Interfaces* **2019**, *11*, 10276-10282.

[25] P.-Q. Liao, X.-W. Chen, S.-Y. Liu, X.-Y. Li, Y.-T. Xu, M. Tang, Z. Rui, H. Ji, J.-P. Zhang, X.-M. Chen, *Chemical Science* **2016**, *7*, 6528-6533.

[26] C. Zhang, W. Song, Q. Ma, L. Xie, X. Zhang, H. Guo, *Energy & Fuels* **2016**, *30*, 4181-4190.

[27] S. Y. W. Chai, L. H. Ngu, B. S. How, *Greenhouse Gases: Science and Technology* **2022**, *12*, 394-427.

[28] A. Momeni, R. V. McQuillan, M. S. Alivand, A. Zavabeti, G. W. Stevens, K. A. Mumford, *Chemical Engineering Journal* **2024**, *480*, 147934.

[29] R. Custelcean, N. J. Williams, K. A. Garrabrant, P. Agullo, F. M. Brethomé, H. J. Martin, M. K. Kidder, *Industrial & Engineering Chemistry Research* **2019**, *58*, 23338-23346.

[30] C.-C. Wei, G. Puxty, P. Feron, *Chemical Engineering Science* **2014**, *107*, 218-226.

[31] F. Zeman, *Environmental Science & Technology* **2007**, *41*, 7558-7563.

[32] Y. E. Kim, J. H. Choi, S. C. Nam, Y. I. Yoon, *Journal of Industrial and Engineering Chemistry* **2012**, *18*, 105-110.

[33] F. A. Chowdhury, H. Yamada, T. Higashii, K. Goto, M. Onoda, *Industrial & Engineering Chemistry Research* **2013**, *52*, 8323-8331.

[34] S.-J. Han, M. Yoo, D.-W. Kim, J.-H. Wee, *Energy & Fuels* **2011**, *25*, 3825-3834.

[35] Q. Guo, C. Chen, Z. Li, X. Li, H. Wang, N. Feng, H. Wan, G. Guan, *Chemical Engineering Journal* **2019**, *371*, 414-423.

[36] M. Damanafshan, B. Mokhtarani, M. Mirzaei, A. Sharifi, *Journal of Molecular Liquids* **2021**, *337*, 116571.

[37] W. Si, S. Ye, D. Zhang, B. Yang, Y. Hou, Z. Li, X. Zhang, J. Zhu, L. Lei, *The Canadian Journal of Chemical Engineering* **2019**, *97*, 697-701.

[38] W. Wang, F. Liu, Q. zhang, G. Yu, S. Deng, *Chemical Engineering Journal* **2020**, *399*, 125734.

[39] J. Wang, M. Wang, W. Li, W. Qiao, D. Long, L. Ling, *AIChE Journal* **2015**, *61*, 972-980.

[40] R. P. Wijesiri, G. P. Knowles, H. Yeasmin, A. F. A. Hoadley, A. L. Chaffee, *Industrial & Engineering Chemistry Research* **2019**, *58*, 3293-3303.

[41] H. T. Kwon, M. A. Sakwa-Novak, S. H. Pang, A. R. Sujan, E. W. Ping, C. W. Jones, *Chemistry of Materials* **2019**, *31*, 5229-5237.

[42] X. Zhu, T. Ge, F. Yang, M. Lyu, C. Chen, D. O'Hare, R. Wang, *Journal of Materials Chemistry A* **2020**, *8*, 16421-16428.

[43] M. Zhao, J. Xiao, W. Gao, Q. Wang, *Journal of Energy Chemistry* **2022**, *68*, 401-410.

[44] Y. Zhang, M. Chen, G. Li, C. Shi, B. Wang, Z. Ling, *Results in Materials* **2020**, *7*, 100102.

[45] N. Lai, Q. Zhu, D. Qiao, K. Chen, L. Tang, D. Wang, W. He, Y. Chen, T. Yu, *Frontiers in Chemistry* **2020**, *8*.

[46] G. Zhang, P. Zhao, L. Hao, Y. Xu, *Journal of CO_2_ Utilization* **2018**, *24*, 22-33.

[47] W. Kong, J. Liu, *New Journal of Chemistry* **2019**, *43*, 6040-6047.

[48] Z. Xue, C. Sun, M. Zhao, Y. Cui, Y. Qu, H. Ma, Z. Wang, Q. Jiang, *ACS Applied Materials & Interfaces* **2021**, *13*, 59834-59842.

[49] Y. Wan, H. Zhou, M. Zheng, Z.-H. Huang, F. Kang, J. Li, R. Lv, *Advanced Functional Materials* **2021**, *31*, 2100300.

[50] T. Wu, X. Zhu, Z. Xing, S. Mou, C. Li, Y. Qiao, Q. Liu, Y. Luo, X. Shi, Y. Zhang, X. Sun, *Angewandte Chemie International Edition* **2019**, *58*, 18449-18453.

[51] Y. Zhao, F. Li, W. Li, Y. Li, C. Liu, Z. Zhao, Y. Shan, Y. Ji, L. Sun, *Angewandte Chemie International Edition* **2021**, *60*, 20331-20341.

[52] X. Wang, M. Luo, J. Lan, M. Peng, Y. Tan, *Advanced Materials* **2021**, *33*, 2007733.

[53] R. Zhao, G. Wang, Y. Mao, X. Bao, Z. Wang, P. Wang, Y. Liu, Z. Zheng, Y. Dai, H. Cheng, B. Huang, *Chemical Engineering Journal* **2022**, *430*, 133085.

[54] H. Wang, Q. Mao, H. Yu, S. Wang, Y. Xu, X. Li, Z. Wang, L. Wang, *Chemical Engineering Journal* **2021**, *418*, 129493.

[55] H. Huang, L. Xia, X. Shi, A. M. Asiri, X. Sun, *Chemical Communications* **2018**, *54*, 11427-11430.

[56] M. W. Chase Jr, *J. Phys. Chem. Ref. Data* **1985**.

[57] G. Herzberg, *Electronic Spectra and Electronic Structure of Polyatomic Molecules*, Van Nostrand, **1966**.

[58] J. M. Plaza, E. Chen, G. T. Rochelle, *AIChE Journal* **2010**, *56*, 905-914.
